# Activation of sphingosine-1-phosphate receptors can relieve myocardial ischemia-reperfusion injury by mitigating oxidative stress and ferroptosis in cardiomyocytes

**DOI:** 10.7150/ijbs.107402

**Published:** 2025-07-28

**Authors:** Xuan Xu, Runqian Li, Shengnan Li, Qin Wei, Fuchao Yu, Genshan Ma, Jiayi Tong

**Affiliations:** 1 Department of Cardiology, Zhongda Hospital, Southeast University, 87 Dingjiaqiao, Nanjing 210009, P.R. China.; 2 School of medicine, Southeast University, Nanjing 210009, P. R. China.

**Keywords:** ferroptosis, reactive oxygen species, sphingosine-1-phosphate receptors, myocardial ischemia, reperfusion injury

## Abstract

**Background:** Myocardial ischemia/reperfusion (MI/R) injury remains a major challenge in cardiovascular therapeutics, with pathogenesis closely associated with reactive oxygen species (ROS) accumulation and ferroptosis. While sphingosine-1-phosphate receptors (S1PRs) activation demonstrates cardioprotective potential against MI/R injury, its mechanistic relationship with redox homeostasis and ferroptotic pathways requires elucidation.

**Methods:** Using hypoxia/reoxygenation (H/R)-treated cardiomyocytes, we investigated S1P-mediated regulation of *Slc7a11*, *Gpx4*, and *MnSOD* transcription through pharmacological inhibition of the S1PRs/Src/STAT3 signaling pathway. Mechanistic insights into S1PRs/Src/STAT3-mediated transcriptional control were obtained through integrated bioinformatics, dual-luciferase reporter assays, chromatin immunoprecipitation, and molecular profiling (qRT-PCR/ Western blotting). In a MI/R mouse model, the therapeutic effects of S1P and Fingolimod were determined using echocardiography, TTC staining, fluorescent probes, and TEM, with mechanisms validated by Western blotting and qRT-PCR.

**Results:** In vitro studies revealed that S1PRs activation (via S1P or Fingolimod) promoted STAT3 phosphorylation and nuclear translocation through Src signaling, thereby enhancing transcriptional upregulation of *Slc7a11*, *Gpx4*, and *MnSOD*. This signaling cascade attenuated H/R-induced ROS generation, mitochondrial damage, and ferroptosis markers, with S1PR1 demonstrating predominant cytoprotection. Chromatin studies confirmed p-STAT3 binding to antioxidant/ferroptosis-related gene promoters. In vivo findings mirrored cellular observations, showing S1PRs agonism significantly improved cardiac function, reduced infarct size, and suppressed myocardial lipid peroxidation compared with untreated controls.

**Conclusions:** Our findings establish that S1PRs signaling confers cardioprotection against MI/R injury through STAT3 phosphorylation-mediated transcriptional activation of antioxidant defense systems and ferroptosis suppression. This mechanistic insight positions S1PRs modulation as a promising therapeutic strategy for ischemic cardiomyopathy.

## Introduction

The rapid restoration of blood flow to the ischemic myocardium has been shown to be the most effective intervention for treating myocardial infarction 1. However, this reperfusion can lead to the reinjury of cardiomyocytes, triggering various forms of cell death and increasing the infarct area, a phenomenon known as myocardial ischemia/reperfusion (MI/R) injury 2. The pathogenic mechanisms underlying MI/R injury are highly complex^3^. Current understanding suggests that oxidative stress, calcium overload, and inflammatory cascades synergistically drive MI/R-related myocardial damage 4. Notably, oxidative stress mitigation through reactive oxygen species (ROS) reduction has demonstrated cardioprotective effects, as excessive ROS generation during reperfusion promotes mitochondrial dysfunction and apoptotic signaling 5. Manganese superoxide dismutase (MnSOD), a mitochondrial antioxidant enzyme, plays a critical role in neutralizing superoxide radicals, with substantial evidence confirming that MnSOD upregulation attenuates MI/R injury severity and improves cardiomyocyte viability 6, 7.

Emerging insights into regulated cell death mechanisms have identified ferroptosis as a key contributor to MI/R pathology 8. This iron-dependent cell death modality, distinct from apoptosis and necrosis, features characteristic lipid peroxidation and ROS accumulation 9. The glutathione peroxidase 4 (GPX4)-glutathione (GSH) axis constitutes the primary defense against ferroptosis, where GPX4 utilizes GSH to reduce cytotoxic lipid hydroperoxides. Compromised *solute carrier family 7 member 11 (SLC7A11*; also called xCT) -mediated cystine uptake impairs GSH synthesis, thereby exacerbating ROS accumulation and ferroptosis 10, 11. Ferroptosis has been established as a crucial target in MI/R injury and can be intervened by regulating ROS generation and the GPX4 pathway 12, 13. As the central transcriptional regulator within the signal transducer and activator of transcription (STAT) family, STAT3 undergoes phosphorylation-dependent activation and nuclear translocation, orchestrating the transcriptional upregulation of anti-ferroptotic and antioxidant genes (e.g., *Slc7a11* and *Gpx4*), which collectively alleviate cardiomyocyte ferroptosis and oxidative stress following MI/R injury 14. Thus, developing targected therapies against these mechanisms represents an urgent clinical need.

Sphingosine-1-phosphate receptors (S1PRs) modulation has emerged as a promising cardioprotective strategy, with multiple studies demonstrating S1PRs activation mitigates myocardial injury 15-18. Endogenous S1P - a bioactive sphingolipid metabolite synthesized by sphingosine kinases - and its structural analog Fingolimod (a clinically approved S1PRs modulator excluding S1PR2) exhibit cardiomyocyte-protective effects through survival pathway activation 19-21. Preclinical studies reveal Fingolimod's AKT-dependent cardioprotection in MI/R models 22, while emerging evidence suggests S1PRs signaling may intersect with ferroptosis and oxidative stress regulatory pathways 17, 18, 23. However, the precise mechanistic relationship between S1PRs activation and ferroptosis regulation in cardiomyocytes remains unexplored.

This investigation provides the first mechanistic evidence that S1P and fingolimod confer cardioprotection against MI/R injury through coordinated modulation of ferroptotic signaling and ROS homeostasis. Through integrated in vivo and in vitro approaches, we systematically elucidate how S1PRs activation preserves cardiomyocyte viability by targeting both ROS and ferroptosis, advancing our understanding of sphingolipid-mediated cardioprotection and identifying potential therapeutic strategies for MI/R injury management.

## Materials and Methods

Isolation of neonatal rat ventricular myocytes (NRVMs) and detailed experimental protocols are provided in the **Supplementary materials**.

### Cardiomyocyte model

The cardiomyocyte hypoxia reoxygenation (H/R) model was prepared as previously described 24. Briefly, cardiomyocytes were initially cultured to 80% confluence. Subsequently, they were cultured in Dulbecco's Modified Eagle's Medium (DMEM) for 24 hours. To induce hypoxia, the cells were cultured in glucose-free and fetal bovine serum (FBS)-free DMEM for 8 hours in a three-gas chamber (37°C, 95% N_2_, and 5% CO_2_). Finally, the medium was replaced with DMEM containing 10% FBS, and cells were transferred to a normoxic incubator (37°C, 95% O_2_, and 5% CO_2_) for 12 hours.

### Animal model

All animal experiments were approved by the Institutional Animal Care and Use Committee of Southeast University (Protocol No. 20230320010) and conducted in compliance with the NIH Guide for the Care and Use of Laboratory Animals. Male C57BL/6 mice (12-week-old) were used to establish the MI/R model. After anesthesia induction with 2-3% isoflurane (1.5 L/min), the chest was shaved and disinfected. A 45-degree oblique incision was made at the point of maximal cardiac impulse. The pectoral muscles were bluntly separated, and a small thoracic incision was created to expose the heart. The left anterior descending (LAD) coronary artery was ligated with a surgical knot, with the suture end externalized. After 45 minutes of ischemia, the ligature was released for reperfusion. Postoperatively, mice were maintained on a warming pad until recovery and monitored daily.

### Statistical analysis

Data analysis was performed using GraphPad Prism 8.0 software (San Diego, CA, USA), and the results are expressed as means with corresponding standard deviations. Student's t test was applied for pairwise comparisons, one-way analysis of variance (ANOVA) was applied for comparisons involving multiple groups, and Tukey's post hoc analysis was applied for multiple group comparisons. p<0.05 was considered statistically significant.

## Results

### 1. S1P alleviates oxidative stress and mitochondrial dysfunction mediated by H/R, thereby reducing cardiomyocyte damage

**Figure 1:**
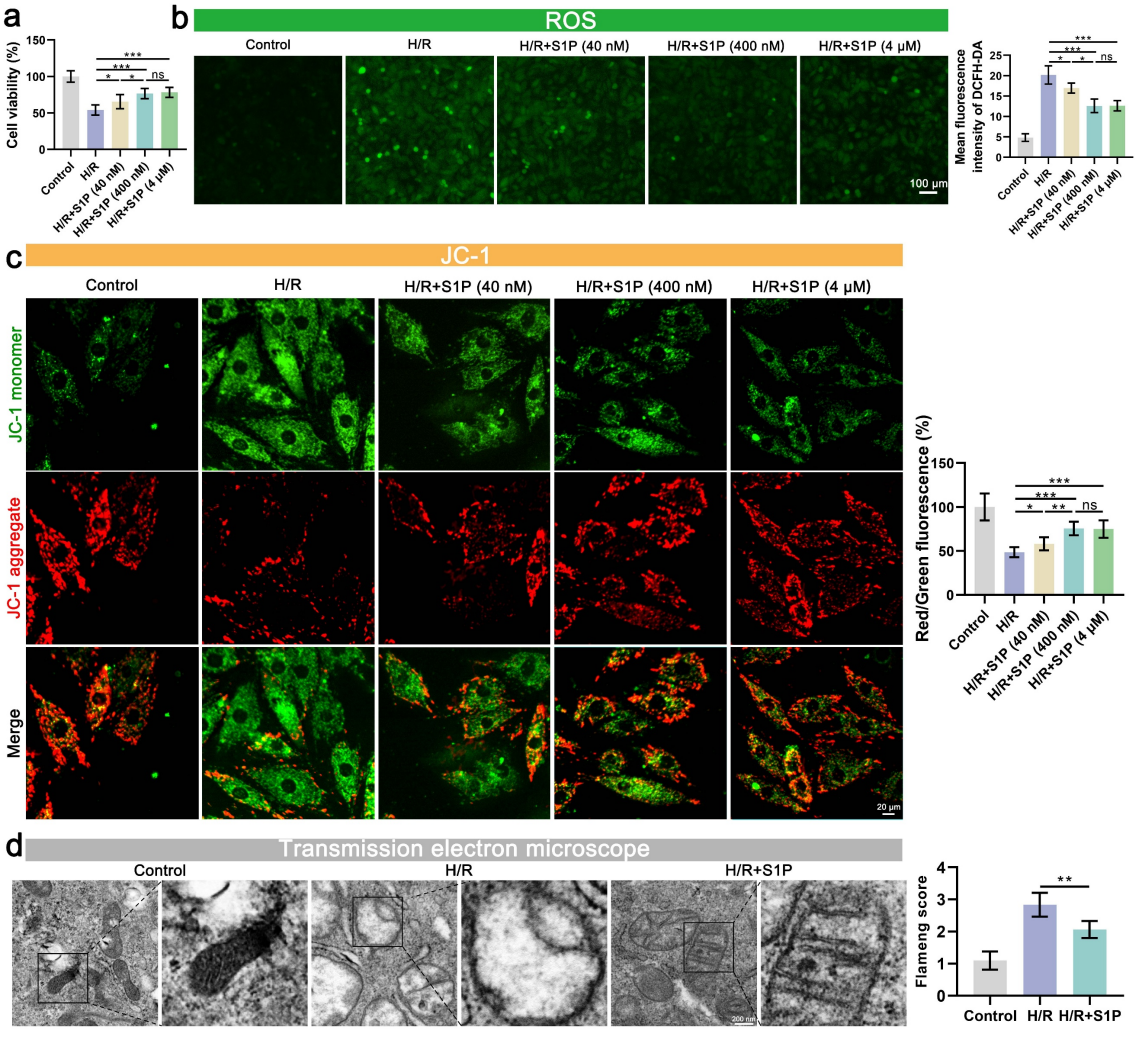
**S1P attenuates H/R-induced oxidative stress, and mitochondrial damage in H9C2 cells.** a. Cell viability assessed by cell counting kit-8 (CCK-8) assay; n=7. b. Measurement of ROS levels in H9C2 cells using 2',7'-dichlorodihydrofluorescein diacetate (DCFH-DA) probe and analysis of mean fluorescence intensity; n=5. c. The MMP was assessed using the JC-1 probe, and the ratio of red to green fluorescence was calculated. Green fluorescence represents JC-1 monomers, while red fluorescence represents JC-1 aggregates; n=5. d. Mitochondrial ultrastructure was analyzed by TEM, and the Flameng score was calculated based on the observed mitochondrial morphology; n=5. Statistical analysis involved one-way ANOVA followed by Tukey's post-hoc test. Lines indicate comparisons between samples, and asterisks denote statistical significance (*P < 0.05, **P < 0.01, ***P < 0.001).

To evaluate the dose-dependent effects of S1P on H/R-induced cardiomyocyte injury, we first performed cell viability assays. H/R treatment significantly reduced cardiomyocyte viability, whereas low- (40 nM), medium- (400 nM), and high-dose (4 μM) S1P treatments all attenuated this effect (**Figure 1a**). Notably, no significant difference was observed between medium- and high-dose groups (**Figure 1a**). Considering that impaired mitochondrial function and oxidative stress are often crucial precipitating factors in decreased cell viability, we further evaluated ROS levels, mitochondrial membrane potential (MMP), and mitochondrial morphology. As shown in **Figure 1b and Figure S1a**, low, medium, and high doses of S1P all mitigated H/R-induced myocardial cell ROS and mitochondrial ROS level. Notably, the medium and high dose S1P treatment groups exhibited the most pronounced inhibition of ROS level and mitochondrial ROS, with no significant difference between the groups (**Figure 1b**). Subsequently, we measured the MMP and adenosine triphosphate (ATP) production (**Figure 1c and Figure S1b**). H/R induced the aggregation of the mitochondrial membrane 5,5',6,6'-tetrachloro-1,1',3,3'-tetraethylbenzimidazolylcarbocyanine iodide (JC-1) towards JC-1 monomers, indicating mitochondrial damage, while S1P treatment helped alleviate this effect. Consistent with previous results, both medium and high dose S1P treatment groups showed the most effectively mitigated MMP decline and attenuated ATP production reduction, with no significant difference between the two groups. Given that there was no significant difference in the therapeutic effects between medium and high dose S1P treatment in cell viability, ROS levels, mitochondrial ROS, MMP, and ATP production experiments, high dose S1P treatment did not confer additional benefits. Therefore, in subsequent experiments, we used medium dose S1P (400 nM) as the optimal concentration for experimentation.

Finally, we examined mitochondrial morphological changes using transmission electron microscopy (TEM). As shown in **Figure 1d**, cellular ultrastructural alterations induced by H/R included an increase in membrane density, membrane rupture, and a decrease in mitochondrial cristae. After S1P treatment, mitochondrial morphological changes were reduced, and Flameng score (a scoring system reflecting mitochondrial damage) evaluation indicated decreased mitochondrial damage.

### 2. S1P can alleviate H/R-induced cardiomyocyte ferroptosis

**Figure 2:**
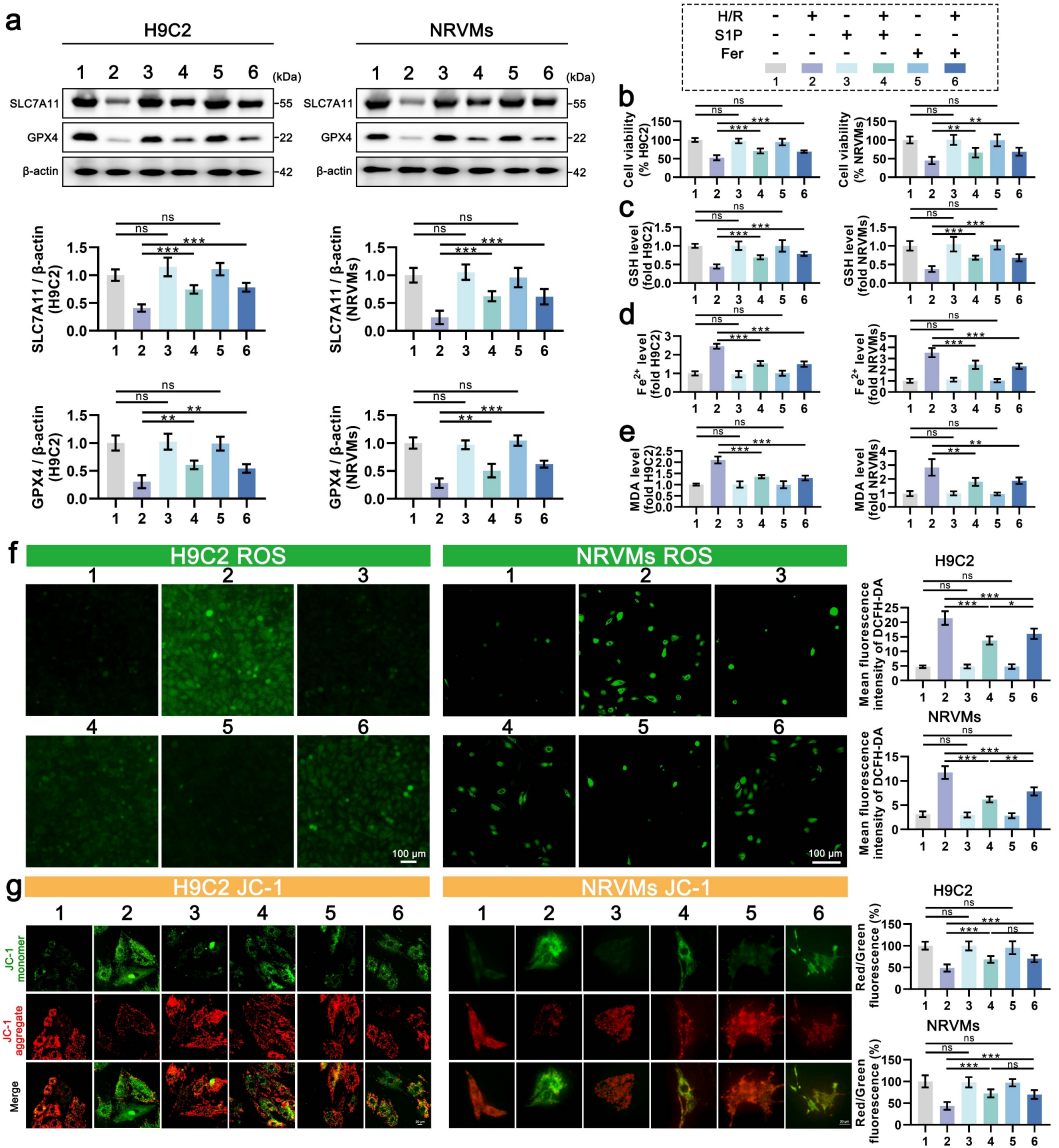
** S1P attenuates ferroptosis and oxidative stress in H9C2 cells and NRVMs.** a. Western blot analysis was performed to assess the protein levels of SLC7A11 and GPX4 in H9C2 cells and NRVMs, and the results were quantitatively analyzed, β-actin loading control; n=6. b. Cell viability of H9C2 cells and NRVMs was assessed using the CCK-8 assay; n=8. c. Determination of GSH levels in H9C2 cells and NRVMs; n=8. d. Determination of Fe²⁺ levels in H9C2 cells and NRVMs; n=8. e. Determination of MDA levels in H9C2 cells and NRVMs; n=8. f. Analysis of ROS levels and mean fluorescence intensity in H9C2 cells and NRVMs was measured using the DCFH-DA probe; n=6. g. MMP in H9C2 cells and NRVMs were assessed using the JC-1 probe, and the ratio of red to green fluorescence was calculated; n=6. All data are means ± standard deviations. Statistical analysis involved one-way ANOVA followed by Tukey's post-hoc test. Lines indicate comparisons between samples, and asterisks denote statistical significance (*P < 0.05, **P < 0.01, ***P < 0.001). Fer = Ferrostatin-1.

Oxidative stress is known to promote lipid peroxidation and mitochondrial dysfunction via excessive ROS production. Given mitochondria's central role in redox homeostasis, their impairment exacerbates oxidative stress and potentiates ferroptosis 9. To determine whether S1P-mediated cardioprotection involves ferroptosis suppression, we analyzed key ferroptotic markers with Ferrostatin-1 as a positive control. As seen in **Figure 2a**, the protein levels of SLC7A11 and GPX4 decreased in cardiomyocytes after H/R treatment but increased after the addition of ferrostatin-1 to inhibit ferroptosis. Similar to ferrostatin-1 treatment, S1P treatment also increased the protein levels of SLC7A11 and GPX4. Consistently, S1P rescued H/R-induced viability loss to levels comparable with Ferrostatin-1 (**Figure 2b**). GSH content is related to the repair of the lipid peroxide system and is involved in the regulation of ferroptosis 11. We observed that S1P reversed H/R-induced GSH reduction with efficacy similar to Ferrostatin-1 (**Figure 2c**). Notably, S1P also attenuated H/R-triggered Fe^2+^ accumulation (a ferroptosis hallmark) and suppressed lipid peroxidation (quantified by malondialdehyde, MDA) to Ferrostatin-1-equivalent levels (**Figure 2d-e**). These data collectively demonstrate S1P's ferroptosis-inhibitory capacity. Intriguingly, S1P exceeded Ferrostatin-1 in suppressing both total ROS and mitochondrial ROS (**Figure 2f and Figure S2a**), despite comparable effects on MMP and ATP restoration (**Figure 2g and Figure S2b**). This divergence suggests that S1P's antioxidant mechanisms extend beyond ferroptosis regulation, potentially involving additional pathways governing redox homeostasis.

### 3. S1P enhances SLC7A11 and MnSOD expression via STAT3 phosphorylation

**Figure 3:**
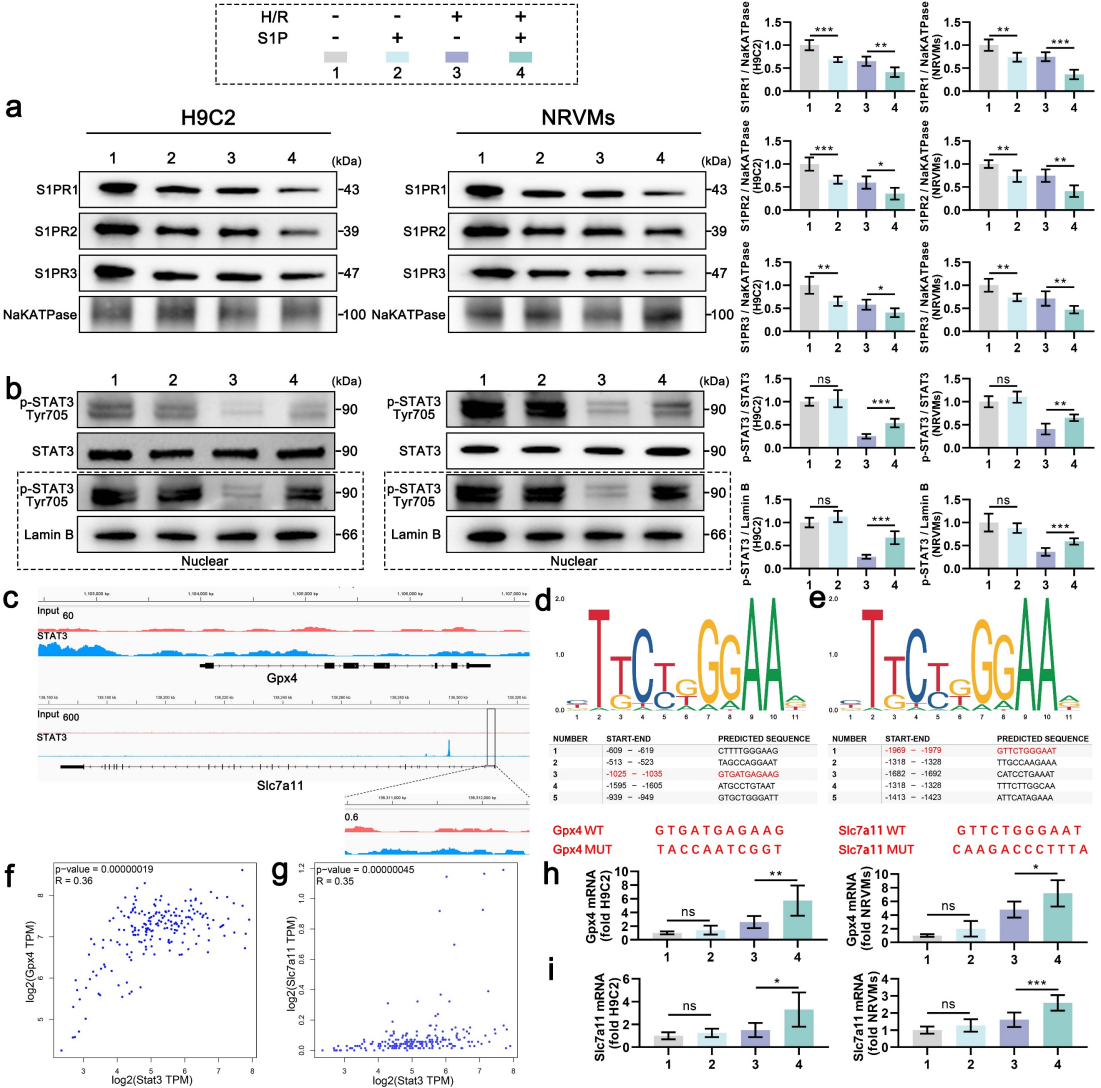
** S1P activates STAT3 signaling to promote the transcription of *Gpx4* and *Slc7a11*.** a. Membrane S1PR1/2/3 levels in H9C2 cells and NRVMs were analyzed by Western blot; NaKATPase loading control; n=6. b. Western blot analysis and quantification of p-STAT3, STAT3 and nuclear p-STAT3 levels in H9C2 cells and NRVMs; LaminB loading control; n=6. c. IGV visualization of STAT3 binding events on the *Gpx4* and *Slc7a11* promoters, derived from ChIP-seq data in the GEO database (GSE117164). d. Schematic of the top five predicted STAT3 binding sites on the *Gpx4* promoter, as predicted by JASPAR, with confirmed binding sites and their respective mutations shown in red. e. Schematic of the top five predicted STAT3 binding sites on the *Slc7a11* promoter, as predicted by JASPAR, with confirmed binding sites and their respective mutations shown in red. f. Pearson correlation analysis of *Stat3* and *Gpx4* mRNA expression in the human left ventricle from the GEPIA database. g. Pearson correlation analysis of *Stat3* and *Slc7a11* mRNA expression in the human left ventricle from the GEPIA database. h. Quantitative analysis of *Gpx4* mRNA expression in H9C2 cells and NRVMs measured by quantitative reverse transcription polymerase chain reaction (qRT-PCR); n=6. i. Quantitative analysis of *Slc7a11* mRNA expression in H9C2 cells and NRVMs measured by qRT-PCR; n=6. All data are means ± standard deviations. Statistical analysis involved one-way ANOVA followed by Tukey's post-hoc test. Lines indicate comparisons between samples, and asterisks denote statistical significance (*P < 0.05, **P < 0.01, ***P < 0.001).

S1PRs activation by S1P triggers tyrosine kinase family Src (Src) kinase phosphorylation, thereby directly enhancing phosphorylation of STAT3 at Tyr705^25^-^27^. Given STAT3's dual role in ferroptosis regulation and redox homeostasis 28, we first assessed Src activation in S1P-treated H9C2 cells. Western blotting confirmed significant p-Src upregulation post-S1P treatment (**Figure S3**). Mechanistically, S1P treatment induced S1PR1/2/3 internalization in both H9C2 cells and NRVMs under normoxic and H/R conditions, indicating receptor-mediated signaling initiation** (Figure 3a)**. Correspondingly, H/R injury suppressed STAT3 phosphorylation, whereas S1P restored phosphorylation signal transducer and activator of transcription 3 (p-STAT3) levels in a Src-dependent manner (**Figure 3b**). Nuclear fractionation assays further revealed enhanced p-STAT3 nuclear translocation upon S1P treatment (**Figure 3b**). Previous ChIP-seq data analysis suggested that STAT3 may interact with the promoters of *Gpx4* and *Slc7a11*, leading to enrichment at these promoter regions (**Figure 3c**). We then used the JASPAR data analysis website to predict potential binding sites between STAT3 and the promoters of *Gpx4* and *Slc7a11*, and listed the five most likely binding sites (**Figure 3d and 3e**). The -1025 to -1035 region in the *Gpx4* promoter and the -1969 to -1979 region in the *Slc7a11* promoter have been shown to interact with STAT3 in tool cell line. Mutation of these sequences (**Figure 3d and 3e**), highlighted in red) suppressed STAT3's ability to enhance *Gpx4* and *Slc7a11* transcription 7, 29. Moreover, *Stat3* mRNA expression positively correlated with *Gpx4* and *Slc7a11* mRNA expression in the mRNA data from human left ventricular tissue in the GEPIA database (**Figure 3f and 3g**). Experimentally, S1P upregulated Gpx4 and Slc7a11 mRNA in parallel with nuclear p-STAT3 accumulation (**Figure 3h and 3i**). These findings establish that S1P/S1PRs/Src signaling enhances STAT3 phosphorylation, facilitating its nuclear translocation to transcriptionally activate Gpx4 and Slc7a11, thereby elevating antioxidant/anti-ferroptosis protein expression.

### 4. S1P enhances antioxidant defense via STAT3-mediated transcriptional activation of *MnSOD*

**Figure 4:**
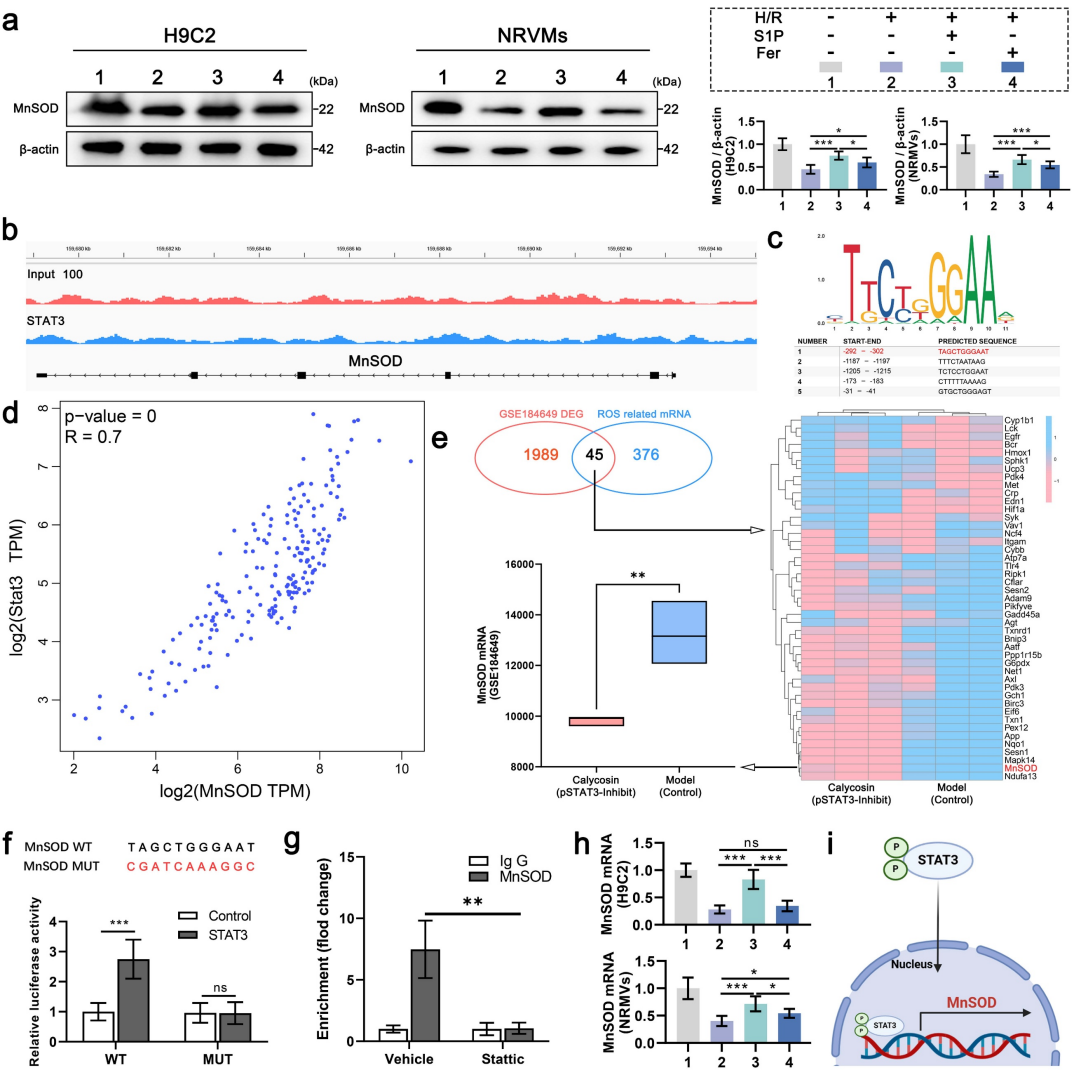
** S1P activates STAT3 signaling to drive *MnSOD* transcription and protein expression.** a. Western blot analysis of MnSOD levels in H9C2 cells and NRVMs, with quantitative analysis; β-actin loading control; n=6. b. IGV view of STAT3 binding events at the *MnSOD* promoter, data from ChIP-seq in the GEO database (GSE212076). c. Schematic of the top five potential STAT3 binding sites in the *MnSOD* promoter predicted by JASPAR. d. Pearson correlation analysis of *Stat3* and *MnSOD* mRNA expression in the human left ventricle from the GEPIA database. e. Heatmap of overlapping DEGs and ROS-related genes in the STAT3 phosphorylation-related dataset (GSE184649); lower left, box plot of *MnSOD* mRNA levels in the dataset. f. Wild-type and mutant *MnSOD* sequences (top); luciferase assay in HEK293T cells overexpressing STAT3 and transfected with reporter plasmids containing WT and MUT *MnSOD* promoters (bottom; n=6). g. AC16 cells treated with vector or Stattic for 48 hours; relative enrichment of STAT3 at the *MnSOD* mRNA promoter was measured by ChIP-qPCR. h. Quantitative analysis of *MnSOD* mRNA expression in H9C2 cells and NRVMs measured by RT-PCR; n=6. i. Schematic of p-STAT3 promoting *MnSOD* transcription in cardiomyocytes. All data are means ± standard deviations. Statistical analysis involved one-way ANOVA followed by Tukey's post-hoc test. Lines indicate comparisons between samples, and asterisks denote statistical significance (*P < 0.05, **P < 0.01, ***P < 0.001). Fer= Ferrostatin-1.

Given that S1P can promote STAT3 phosphorylation, which has been proven to enhance MnSOD protein levels and regulate cellular ROS levels^30^. To determine the effect of increased p-STAT3 on MnSOD and explore the ROS level differences caused by S1P and Ferrostatin-1 treatments. MnSOD protein levels were measured in H9C2 cells and NRVMs after S1P and Ferrostatin-1 treatment, showing that S1P significantly upregulated MnSOD protein levels in H9C2 cells and NRVMs, exceeding Ferrostatin-1's effects (**Figure 4a**). The results above led us to contemplate whether the mechanism involved is similar to that of *Gpx4* and *Slc7a11* transcriptional upregulation, wherein p-STAT3 enters the nucleus to enhance *MnSOD* transcription, resulting in elevated MnSOD protein level. Therefore, we analyzed the relevant ChIP-seq dataset, which showed an enrichment of STAT3 at the *MnSOD* promoter site (**Figure 4b**). We then used the JASPAR database to predict potential *MnSOD* promoter binding sites, with promoter site 1 having the highest binding score of 0.91 (**Figure 4c**). GEPIA database analysis showed a significant positive correlation between *Stat3* and *MnSOD* mRNA expression in human left ventricular tissue (**Figure 4d**). Additionally, we analyzed a transcriptomic sequencing dataset related to STAT3 phosphorylation in rat cardiomyocytes, where p-STAT3 was inhibited in the Calycosin group. In this dataset, 2034 differentially expressed genes (DEGs) were identified and compared with 421 ROS-related genes, revealing 45 overlapping genes, notably including *MnSOD*. Moreover, *MnSOD* mRNA expression was downregulated in cardiomyocytes following p-STAT3 inhibition (**Figure 4e**). The above evidence indicates that p-STAT3 can enhance *MnSOD* transcription in cardiomyocytes. Therefore, we mutated the *MnSOD* promoter and cloned it into a dual-luciferase reporter construct, followed by a luciferase reporter assay. The results showed that STAT3 protein overexpression increased wild-type *MnSOD* promoter activity but did not enhance mutant *MnSOD* promoter activity (**Figure 4f**). We also performed ChIP-qPCR analysis, which showed that STAT3 binds to the promoter of the *MnSOD* gene in cardiomyocytes (**Figure 4 g**). Finally, we confirmed that S1P treatment significantly upregulates *MnSOD* mRNA expression in H9C2 cells and NRVMs (**Figure 4h**). In summary, after phosphorylation, STAT3 translocates into the nucleus in cardiomyocytes and then accumulates at the *MnSOD* promoter region, promoting *MnSOD* transcription (**Figure 4i**). Thus, the ability of S1P to upregulate MnSOD protein levels is conferred by STAT3 signaling activation.

### 5. STAT3 signaling is essential for S1P-mediated anti-ferroptosis and antioxidant effects

**Figure 5:**
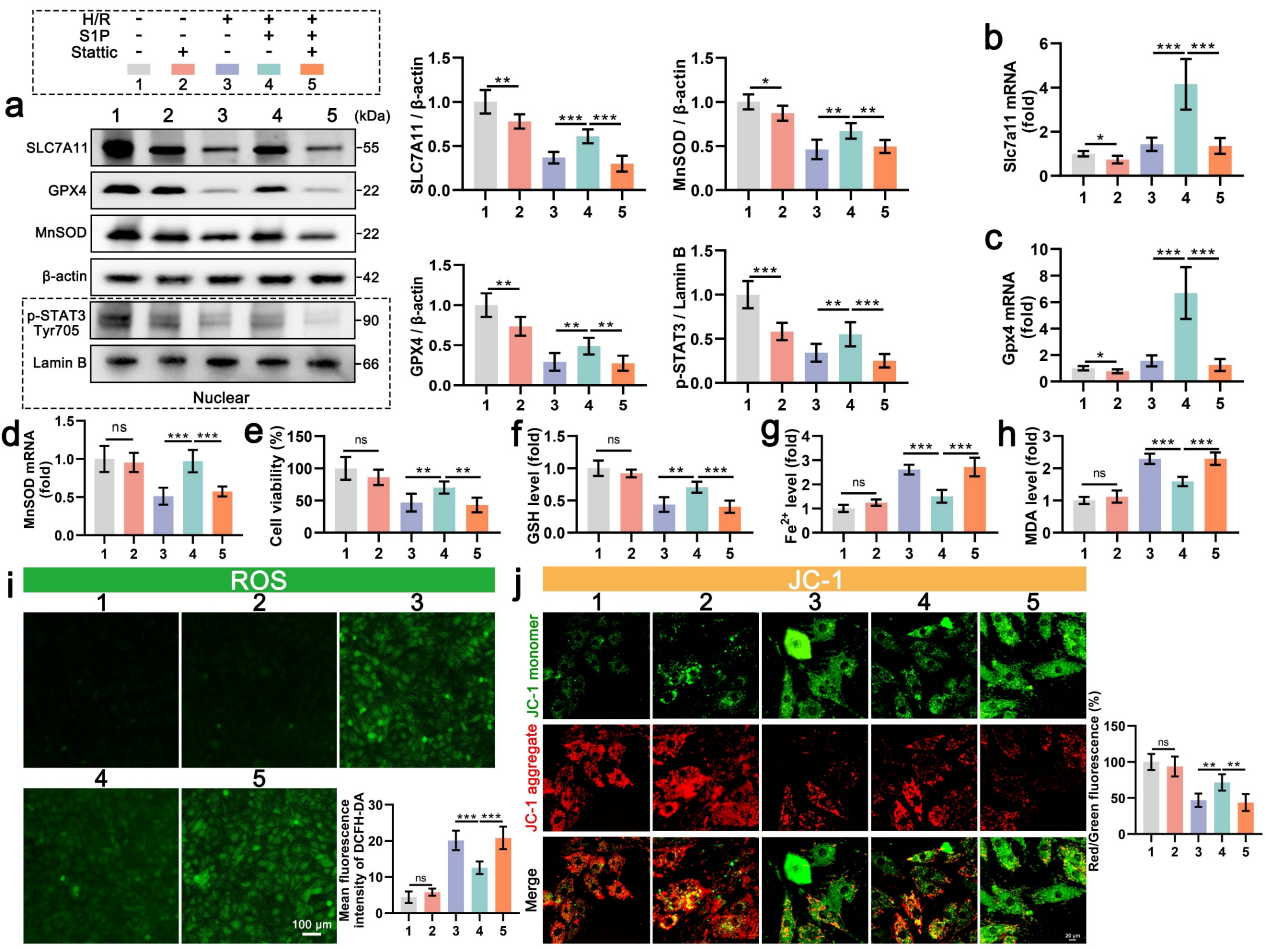
** STAT3 inhibition abrogates S1P-mediated anti-ferroptosis and antioxidant effects in H9C2 cells.** a. The levels of SLC7A11, GPX4, and MnSOD, along with nuclear p-STAT3, were analyzed using Western blot in H9C2 cells, with quantification of results; β-actin and LaminB loading control; n=6. b. The expression of *Slc7a11* mRNA in H9C2 cells was quantified using RT-PCR; n=6. c. The expression of *Gpx4* mRNA in H9C2 cells was quantified using RT-PCR; n=6. d. The expression of *MnSOD* mRNA in H9C2 cells was quantified using QRT-PCR; n=6. e. H9C2 cell viability was evaluated using the CCK-8 assay; n=8. f. Determination of GSH content in H9C2 cells; n=8. g. Determination of Fe^2+^ content in H9C2 cells; n=8. h. Determination of MDA content in H9C2 cells; n=8. i. Measurement of ROS levels in H9C2 cells using DCFH-DA probe and analysis of mean fluorescence intensity; n=5. j. MMP in H9C2 cells were assessed using the JC-1 probe, and the ratio of red to green fluorescence was calculated; n=6. All data are means ± standard deviations. Statistical analysis involved one-way ANOVA followed by Tukey's post-hoc test. Lines indicate comparisons between samples, and asterisks denote statistical significance (*P < 0.05, **P < 0.01, ***P < 0.001).

To further confirm the role of STAT3 signaling in the regulation of S1P mediated ferroptosis, we employed Stattic (a STAT3 signaling inhibitor) to suppress STAT3 phosphorylation. Nuclear fractionation confirmed significant p-STAT3 reduction in Stattic-treated cardiomyocytes (**Figure 5a**). Concomitantly, S1P failed to rescue H/R-induced downregulation of SLC7A11, GPX4, and MnSOD proteins (**Figure 5a**), with corresponding suppression of *Slc7a11*, *Gpx4*, and *MnSOD* mRNA in cardiomyocytes (**Figure 5b, 5c, and 5d**). Additionally, we evaluated the effect of Stattic on cardiomyocyte viability after S1P treatment. Results indicated that inhibition of STAT3 signaling diminished S1P's capacity to restore cardiomyocyte viability post H/R treatment (**Figure 5e**). Furthermore, when STAT3 signaling is inhibited, S1P's regulatory effects on GSH, Fe^2+^, and MDA in H/R-treated cardiomyocytes are weakened (**Figure 5f, 5g, and 5h**). These results indicate that when STAT3 phosphorylation is suppressed, S1P's capacity to counteract H/R-induced ferroptosis in cardiomyocytes is diminished. Lastly, we evaluated oxidative stress, ATP production and MMP in cardiomyocytes across all groups. Consistent with expectations, when STAT3 signaling is suppressed, the mitochondrial ROS and cell ROS inhibiting effect of S1P in cardiomyocytes is reduced (**Figure 5i and Figure S4a**), and mitochondrial protective ability decreases (**Figure 5j and Figure S4b**). Overall, when STAT3 signaling is inhibited, S1P's ability to reverse H/R-induced cellular ferroptosis and ROS effects is reduced, highlighting the critical role of STAT3 signaling in S1P-mediated ferroptosis and ROS regulation in cardiomyocytes.

### 6. S1P primarily exerts its inhibitory effects on ferroptosis and ROS through the activation of S1PR1

**Figure 6:**
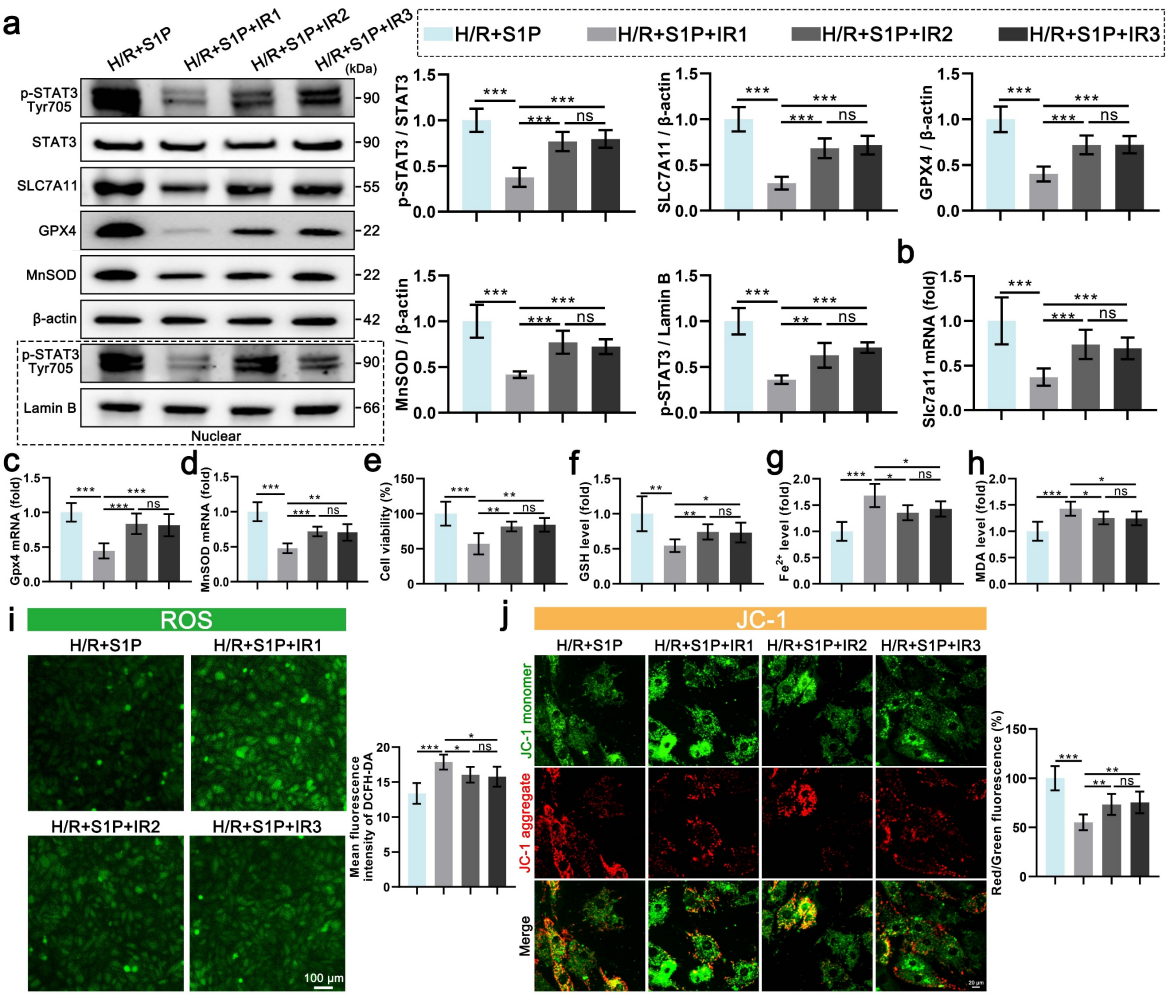
** Inhibition of S1PR1/2/3 signaling attenuates S1P-mediated ferroptosis and oxidative stress regulation in H9C2 cells.** a. The levels of p-STAT3, STAT3, SLC7A11, GPX4, and MnSOD, along with nuclear p-STAT3, were analyzed using Western blot in H9C2 cells, with quantification of results; β-actin and LaminB loading control; n=5. b. The expression of *Slc7a11* mRNA in H9C2 cells was quantified using qRT-PCR; n=5. c. The expression of *Gpx4* mRNA in H9C2 cells was quantified using qRT-PCR; n=5. d. The expression of *MnSOD* mRNA in H9C2 cells was quantified using qRT-PCR; n=5. e. H9C2 cell viability was evaluated using the CCK-8 assay; n=6. f. Determination of GSH content in H9C2 cells; n=6. g. Determination of Fe^2+^ content in H9C2 cells; n=6. h. Determination of MDA content in H9C2 cells; n=6. i. Measurement of ROS levels in H9C2 cells using DCFH-DA probe and analysis of mean fluorescence intensity; n=5. j. MMP assessed by JC-1 probe; results are presented as the ratio of red/green fluorescence; n=5. All data are means ± standard deviations. Statistical analysis involved one-way ANOVA followed by Tukey's post-hoc test. Lines indicate comparisons between samples, and asterisks denote statistical significance (*P < 0.05, **P < 0.01, ***P < 0.001). IR1= W146; IR2= JTE013; IR3=CAY10444.

S1P exerts its effects by interacting with three types of S1PRs on the membranes of cardiomyocytes (S1PR1/2/3), which all promote the phosphorylation of STAT3^26^, ^31^, ^32^. To further explore the mechanism of S1P, we separately inhibited S1PR1/2/3, evaluating their effects on STAT3 phosphorylation in cardiomyocytes and their impact on the transcriptional regulation of *Gpx4*, *Slc7a11*, and *MnSOD*. As shown in **Figure 6a**, when S1PR1 signaling is inhibited, the ratio of p-STAT3 to STAT3 decreases by over 62.43%. In comparison, inhibition of S1PR2 or S1PR3 signaling decreases the ratios of p-STAT3 to STAT3, respectively. Subsequent detection of nuclear p-STAT3 protein also indicated that the nuclear translocation of p-STAT3 decreases with the inhibition of S1PR1/2/3 signaling. Among these, the inhibition of S1PR1 resulted in the most significant reduction in the nuclear translocation of p-STAT3 (**Figure 6a**). Similarly, S1PR1 inhibition led to the most significant decreases in SLC7A11, GPX4, and MnSOD protein levels in cardiomyocytes (**Figure 6a**). While S1PR2 or S1PR3 inhibition also reduced these proteins, the effects were less marked compared to S1PR1 inhibition (**Figure 6a**). The mRNA expression of *Gpx4*, *Slc7a11*, and *MnSOD* parallels the changes in protein levels, with S1PR1 inhibition inducing the strongest transcriptional suppression (**Figure 6b, 6c, and 6d**). In contrast, S1PR2/S1PR3 inhibition had minimal effects on mRNA downregulation (**Figure 6b, 6c, and 6d**). To assess the roles of S1PR1/2/3 in ferroptosis and oxidative stress, we measured cell viability, GSH, Fe^2+^, MDA, and ROS levels in each group. S1PR1 inhibition caused the most severe decline in cardiomyocyte viability (**Figure 6e**), along with greater sensitivity in GSH depletion, Fe^2+^ accumulation, and MDA elevation (**Figure 6f, 6g, and 6h**).

Finally, oxidative stress markers, ATP production, and MMP were analyzed. S1PR1 inhibition resulted in the highest cellular/mitochondrial ROS levels and reduced MMP and ATP production, whereas S1PR2/S1PR3 inhibition exhibited significantly weaker effects on these parameters (**Figure 6i, 6j and Figure S5a, S5b**). These findings demonstrate that S1P regulates STAT3 signaling in cardiomyocytes via S1PR1/2/3, mediating ferroptosis and oxidative stress, with S1PR1 playing the dominant role in this pathway.

### 7. Fingolimod can also alleviate ferroptosis and oxidative stress in cardiomyocytes after H/R via S1PR-dependent STAT3 activation

**Figure 7:**
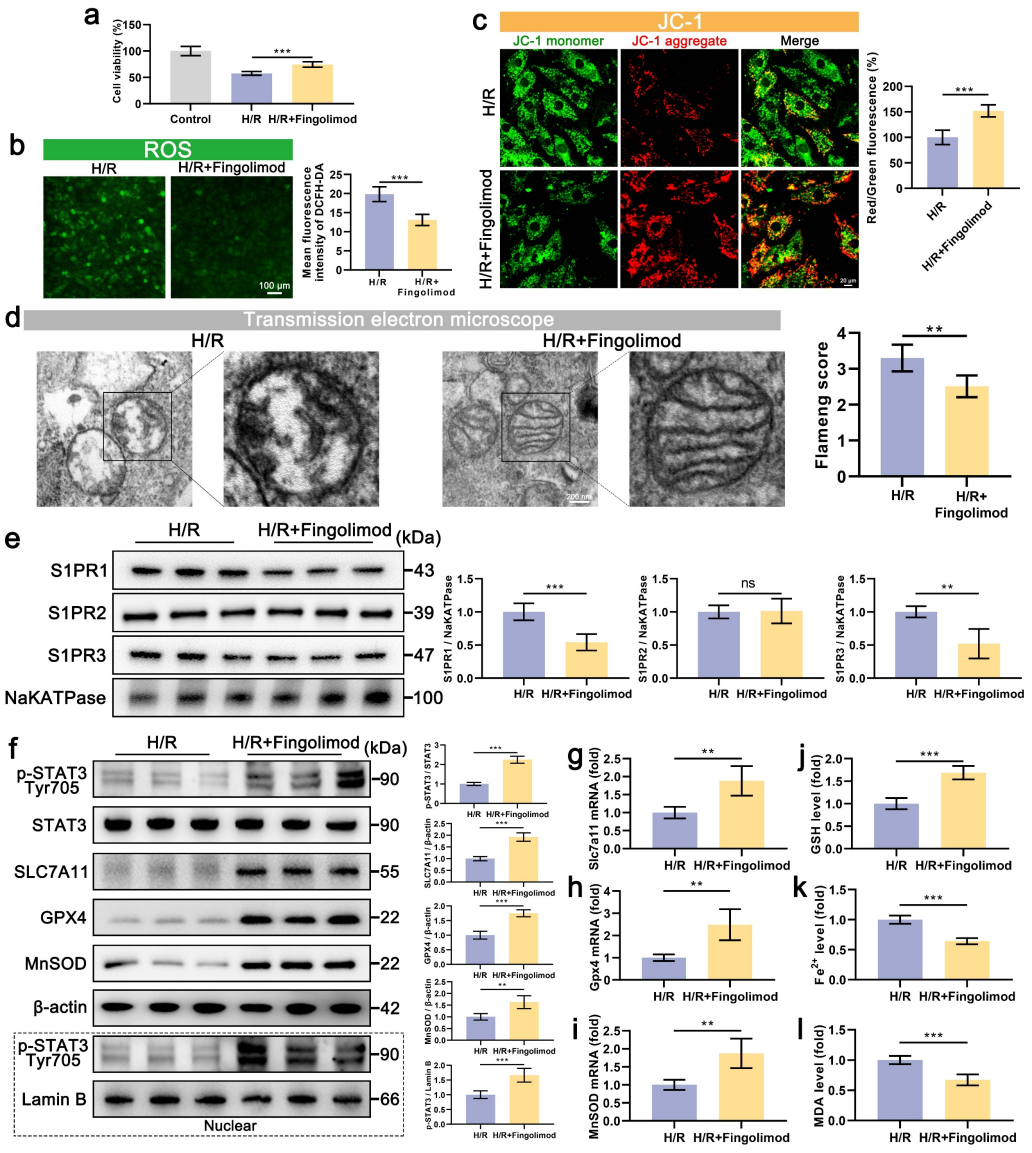
** Fingolimod alleviates cardiomyocyte damage induced by H/R.** a. Cell viability assessed by CCK-8 assay; n=8. b. Measurement of ROS levels in H9C2 cells using DCFH-DA probe and analysis of mean fluorescence intensity; n=5. c. The MMP was assessed using the JC-1 probe, and the ratio of red to green fluorescence was calculated. Green fluorescence represents JC-1 monomers, while red fluorescence represents JC-1 aggregates; n=5. d. The mitochondrial morphology was examined using TEM, and the Flameng score was calculated based on the observed mitochondrial morphology; n=5. e. Western blot analysis and quantification of S1PR1, S1PR2, and S1PR3 levels in the membranes of H9C2 cells; NaKATPase loading control; n=5. f. The levels of p-STAT3, STAT3, SLC7A11, GPX4, and MnSOD, along with nuclear p-STAT3, were analyzed using Western blot in H9C2 cells, with quantification of results; β-actin and LaminB loading control; n=5. g. The expression of* Slc7a11* mRNA in H9C2 cells was quantified using qRT-PCR; n=5. h. The expression of* Gpx4* mRNA in H9C2 cells was quantified using qRT-PCR; n=5. i. The expression of *MnSOD* mRNA in H9C2 cells was quantified using qRT-PCR; n=5. j. Determination of GSH content in H9C2 cells; n=5. k. Determination of Fe^2+^ content in H9C2 cells; n=5. l. Determination of MDA content in H9C2 cells; n=5. All data are means ± standard deviations. Statistical analysis involved one-way ANOVA followed by Tukey's post-hoc test. Lines indicate comparisons between samples, and asterisks denote statistical significance (*P < 0.05, **P < 0.01, ***P < 0.001).

Our findings demonstrate that S1P alleviates H/R-induced ferroptosis and oxidative stress in cardiomyocytes by activating S1PR1/2/3 and downstream STAT3 signaling. Notably, the S1P analog Fingolimod, which has been FDA-approved and clinically used for multiple sclerosis treatment, can also activate S1PR. The results show that Fingolimod can enhance the viability of cardiomyocytes treated with H/R (**Figure 7a**). Further experiments on oxidative stress and mitochondrial phenotype indicate that Fingolimod can reduce H/R-induced cell ROS and mitochondrial ROS production (**Figure 7b and Figure S6a**), decrease in MMP and ATP production levels (**Figure 7c and Figure S6b**), and abnormal mitochondrial morphology in cardiomyocytes (**Figure 7d**). Mechanistically, fingolimod binds to S1PR1/3, inducing receptor internalization (**Figure 7e**), thereby activating a Src kinase signaling cascade (**Figure S3**) that in turn enhances phosphorylation of STAT3 at tyrosine 705 (**Figure 7f**). This was accompanied by enhanced nuclear translocation of p-STAT**3 (Figure 7f**), which upregulated the transcription of *Gpx4*, *Slc7a11*, and *MnSOD* (**Figure 7g, 7h, and 7i**), and upregulating the protein levels of SLC7A11, GPX, and MnSOD (**Figure 7f**). Finally, we assessed ferroptosis indicators, and Fingolimod increased GSH content (**Figure 7j**) while decreasing Fe^2+^ and MDA accumulation (**Figure 7k and 7l**). Similar to S1P, fingolimod activates the STAT3 pathway via S1PRs, attenuating ferroptosis, oxidative stress, and mitochondrial dysfunction in H/R-injured cardiomyocytes.

### 8. S1P and Fingolimod alleviate myocardial damage and cardiac dysfunction induced by MI/R

**Figure 8:**
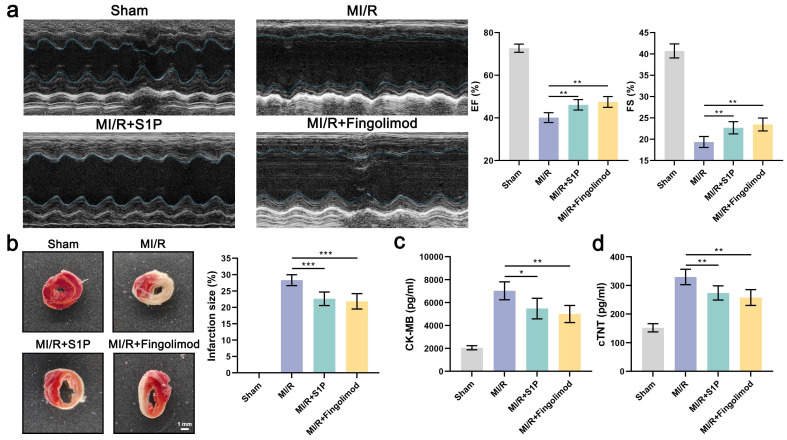
** S1P and Fingolimod can reverse cardiac damage and cardiac function in MI/R mice.** a. Representative echocardiograms, ejection fraction (EF) and fractional shortening (FS) calculated from echocardiograms; n=5. b. Myocardial tissue infarct size revealed by TTC staining in MI/R mice; n=5. c. Serum CK-MB levels in all groups; n=5. d. Serum cTNT levels in all groups; n=5. All data are means ± standard deviations. Statistical analysis involved one-way ANOVA followed by Tukey's post-hoc test. Lines indicate comparisons between samples, and asterisks denote statistical significance (*P < 0.05, **P < 0.01, ***P < 0.001).

In vitro experiments demonstrated that both S1P and fingolimod suppress ferroptosis in cardiomyocytes, mitigating mitochondrial dysfunction, oxidative stress, and cell death. To validate these findings in vivo, we evaluated the cardioprotective effects of S1P and fingolimod in a mice model of MI/R. Echocardiographic analysis revealed that S1P and fingolimod treatment significantly improved EF and FS in MI/R mice (**Figure 8a**). Subsequently, we assessed the impact of S1P and Fingolimod on the infarct area using 2,3,5-triphenyltetrazolium chloride (TTC) staining. As depicted in **Figure 8b**, both S1P and Fingolimod reduced the myocardial infarct area in MI/R mice. Finally, we measured the cardiac injury markers creatine kinase-myocardial band (CK-MB) and cardiac troponin T (cTNT), consistent with previous findings, demonstrating that S1P and Fingolimod can decrease CK-MB and cTNT levels (**Figure 8c and 8d**). Collectively, these results demonstrate that S1P and fingolimod alleviate cardiac dysfunction, limit myocardial damage, and improve functional recovery following MI/R injury.

### 9. S1P and Fingolimod attenuate ferroptosis and oxidative stress in MI/R injury via STAT3 activation

**Figure 9:**
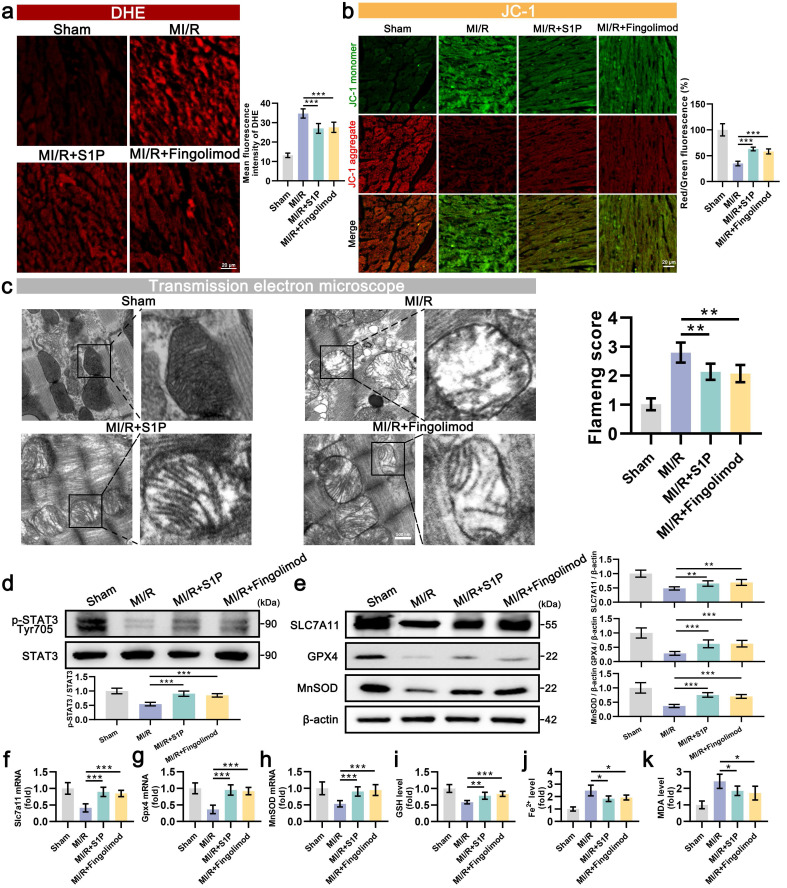
** S1P and Fingolimod can reduce oxidative stress, ferroptosis, and mitochondrial dysfunction in the left ventricular tissue of MI/R mice.** a. Measurement of oxidative stress levels in heart tissue using dihydroethidium (DHE) probe and analysis of mean fluorescence intensity; n=5. b. The MMP was assessed using the JC-1 probe, and the ratio of red to green fluorescence was calculated. Green fluorescence represents JC-1 monomers, while red fluorescence represents JC-1 aggregates; n=5. c. The mitochondrial morphology was examined using transmission electron microscopy, and the Flameng score was calculated based on the observed mitochondrial morphology; n=5. d. The levels of p-STAT3 and STAT3 were analyzed using Western blot in left ventricular tissue, with quantification of results; n=6. e. The levels of SLC7A11, GPX4, and MnSOD were analyzed using Western blot in left ventricular tissue, with quantification of results; β-actin loading control; n=6. f. Quantify the expression of *Slc7a11* mRNA in the left ventricular tissue of mice using qRT-PCR; n=6. g. Quantify the expression of *Gpx4* mRNA in the left ventricular tissue of mice using qRT-PCR; n=6. h. Quantify the expression of *MnSOD* mRNA in the left ventricular tissue of mice using qRT-PCR; n=6. i. GSH levels determination results; n=5. j. Fe^2+^ content measurement results, n=5. k. MDA content measurement results; n=5. All data are means ± standard deviations. Statistical analysis involved one-way ANOVA followed by Tukey's post-hoc test. Lines indicate comparisons between samples, and asterisks denote statistical significance (*P < 0.05, **P < 0.01, ***P < 0.001).

Subsequent assessments of oxidative stress and MMP in cardiac tissues. The results indicated that in the MI/R mouse model, S1P and Fingolimod treatment reduced ROS levels (**Figure 9a**) and alleviated damage to cardiac mitochondrial membrane potential (**Figure 9b**). TEM images of the injured area revealed significant vacuolization of mitochondria in cardiomyocytes of MI/R mice, along with blurred and ruptured mitochondrial cristae. Treatment with S1P and Fingolimod alleviated the vacuolization and rupture of mitochondrial cristae in the heart (**Figure 9c**). The consistency of phenotypic and functional findings between in vitro and in vivo models prompted further mechanistic validation. Western blot results indicated that S1P and Fingolimod promote STAT3 phosphorylation (**Figure 9d**) and upregulate the protein levels of SLC7A11, GPX4 and MnSOD (**Figure 9e**). Additionally, the expression of *Slc7a11*,* Gpx4*, and *MnSOD* mRNA in cardiac tissue is consistent with the trends observed in protein levels (**Figure 9f, 9g and 9h**). Finally, ferroptosis-related assays were conducted, confirming that S1P and Fingolimod treatment increased GSH levels (**Figure 9i**) and reduced the accumulation of Fe^2+^ and MDA in the left ventricular tissue of MI/R mice (**Figure 9j and 9k**). In conclusion, the results of in vivo experiments validate our in vitro findings, demonstrating that S1P and fingolimod can alleviate oxidative stress and ferroptosis, thereby mitigating mitochondrial dysfunction. This ultimately leads to a reduction in cardiomyocyte damage and an improvement in cardiac function in MI/R mice.

## Discussion

In recent decades, the incidence of myocardial ischemia has been steadily increasing. Although reperfusion therapy can restore blood flow, it may also lead to more severe injury. During reperfusion, when blood flow reintroduces oxygen into cardiac tissue, ROS and other oxidative stressors are concomitantly generated. Intracellular danger factors activate both extrinsic and intrinsic apoptotic pathways, resulting in elevated levels of cleaved caspase-3 33. When the stimulus is sufficiently intense, necroptosis occurs 33. Meanwhile, iron metabolism is disrupted and the antioxidant system is impaired due to exacerbated oxidative stress, leading to an imbalance in intracellular redox status and lipid metabolism, which triggers ferroptosis 9, 12. While ferroptosis, apoptosis, and necroptosis exhibit distinct characteristics, crosstalk exists between them. In cardiomyocytes, ferroptosis can trigger apoptosis via endoplasmic reticulum stress, and mitigating ferroptosis can partially alleviate apoptosis 34. Therefore, exploring drugs targeting cardiomyocyte ferroptosis and oxidative stress holds significant importance for the clinical treatment of MI/R injury.

S1P plays a crucial role in cardiovascular physiology 35. Studies have indicated decreased levels of S1P in the circulation of patients with coronary artery disease and myocardial infarction 36, 37. Translational research has shown associations between plasma S1P and various cardiovascular disease markers 38. S1P has been demonstrated to protect mitochondrial function in the heart, with research suggesting its role in preventing MI/R injury through AKT / GSK3β phosphorylation^15^. Previous studies have hinted at the mitochondrial protective function of S1P being linked to the STAT3 signal, although the specific mechanisms were not elaborated 39. Fingolimod has long been considered a potential therapeutic agent for ischemic diseases, with extensive research indicating its favorable treatment outcomes for stroke. Notably, Fingolimod has been employed in at least two clinical trials related to stroke, underscoring its potential unique role in the treatment of ischemic conditions 40. S1PRs are a group of G protein-coupled receptors (GPCR), consisting of five isoforms (S1PR1-S1PR5), that serve as targets for the lipid signaling molecule S1P 41. The levels of these receptors vary in different organs, and S1P exerts various biological functions by activating these receptors 41. There are three types of S1PRs (S1PR1/2/3) in cardiomyocytes, with S1PR1 protein level significantly higher than that of S1PR2/3 42. Numerous studies have established that S1P exerts cardioprotective effects by activating cardiomyocyte S1PRs. S1PR1 activation can improve cardiac function and myocardial healing after myocardial infarction in mice 43. S1PR1 signaling activation can reduce H/R-induced cardiomyocyte apoptosis through the cAMP / AKT pathway 44. In the transverse aortic constriction model, S1PR2 / ERK pathway activation plays an anti-remodeling role 45. Conditional S1PR3 knockdown aggravates heart function damage and increases infarct size in MI/R mice 46.

The molecular mechanism by which S1PRs regulate STAT3 phosphorylation involves two key effectors of GPCR signaling pathways: G proteins and β-arrestins family proteins 47, 48. Biochemical studies reveal that upon binding to β-arrestins 49-52, S1PRs undergo conformational rearrangements to assemble a Src kinase recruitment complex, where allosteric activation of the G protein subunits triggers Src signaling cascades 53, 54. The phosphorylated/activated Src subsequently catalyzes site-specific phosphorylation of STAT3 at tyrosine 705, a post-translational modification that functions as a molecular switch governing STAT3 nuclear translocation and transcriptional activity 55, 56. Notably, S1PR1/2/3 subtypes have been demonstrated to directly regulate the STAT3 signaling axis through this Src-dependent phosphorylation pathway 25-27, suggesting potential subtype conservation of this regulatory mechanism within the S1PR family. Previous investigations have shown that activation of S1PR1/2/3 signaling enhances STAT3 phosphorylation to mediate biological effects 26, 31, 32, 57, 58. In this study, we confirmed that S1P activates S1PR1/2/3 to promote Src activation in cardiomyocytes, thereby increasing STAT3 phosphorylation. Consistent with this, fingolimod was found to enhance Src-mediated STAT3 phosphorylation in cardiomyocytes through activation of S1PR1/3.

STAT3 is a transcription factor that mediates the expression of multiple genes and plays a pivotal role in oxidative stress and apoptosis in various cell processes. Studies have shown that activation of STAT3 plays a crucial protective role in the heart following myocardial ischemia 59. STAT3 helps restore mitochondrial function in cardiomyocytes after MI/R through mechanisms such as reducing the production of mitochondrial reactive oxygen species and decreasing the opening of the mitochondrial permeability transition pore 60. Previous studies have confirmed that phosphorylated STAT3 can translocate to the nucleus, bind to the promoter regions of *Gpx4* and *Slc7a11*, and promote their transcription 28, 61. Studies have shown that increasing the levels of GPX4 and SLC7A11 can inhibit ferroptosis in mouse cardiomyocytes induced by H/R 62. In this study, we observed that elevated STAT3 phosphorylation in cardiomyocytes promotes the transcription of Gpx4 and Slc7a11, thereby increasing GPX4 and SLC7A11 protein expression levels, which ultimately attenuates ferroptosis in cardiomyocytes. Notably, in this study, we found that S1P exhibits superior antioxidant effects compared to Ferrostatin-1. Based on the comprehensive findings, we attribute this to S1P promoting MnSOD expression via the STAT3 signaling pathway, which endows S1P with an antioxidant signaling axis independent of ferroptosis. Early studies have shown that constitutively activated STAT3 (often referred to as p-STAT3) can increase MnSOD expression in cardiomyocytes, thereby providing protection against H/R-induced damage 30. Furthermore, in myocardial infarction models, STAT3 can co-localize with MnSOD to increase its activity 63. Although there is substantial evidence that STAT3 promotes MnSOD transcription in cardiomyocytes, further in-depth studies have not been conducted. Prior studies have indicated that STAT3 can bind to bases within the mouse brain tissue MnSOD promoter regions -557 to -830 and -213 to -556, but the specific binding sites of the MnSOD promoter have not been accurately identified 64. In our study, we observed the ChIP-seq dataset and validated the binding of STAT3 to the MnSOD promoter through dual-luciferase and ChIP-qPCR experiments.

**Figure 10:**
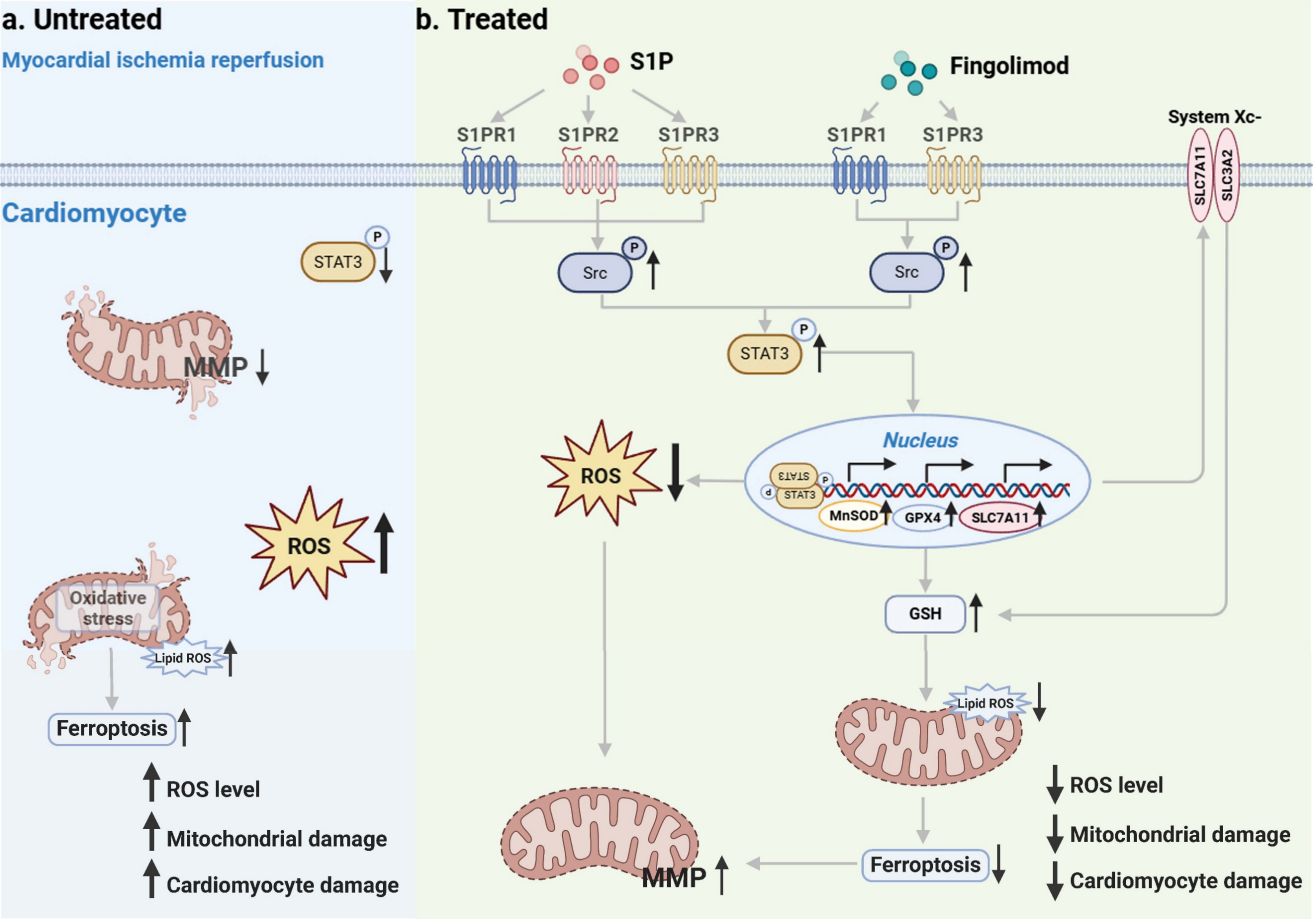
**Schematic.** a. Following MI/R, the occurrence of intracellular ROS and ferroptosis leads to mitochondrial damage, ultimately resulting in cardiomyocyte injury. b. S1P and its analog fingolimod specifically activate S1PRs to initiate the Src/STAT3 signaling cascade. Additionally, it translocates to the nucleus, enhancing the transcription of *Slc7a11*, *Gpx4*, and *MnSOD*, thereby regulating GSH, lipid peroxides and ROS. The combined suppression of ROS and ferroptosis alleviates mitochondrial damage, consequently mitigating cardiomyocyte injury.

In this study, we conducted both in vivo and in vitro experiments using MI/R mice and H/R cardiomyocytes, respectively. Our findings demonstrate that S1P and Fingolimod alleviate mitochondrial damage by reducing ROS and ferroptosis, ultimately suppressing cardiomyocyte death and cardiac injury induced by MI/R. Mechanistically, after MI/R injury occurs in cardiomyocytes, p-STAT3 levels decrease, ROS levels increase, accompanied by the occurrence of ferroptosis (**Figure 10a**). S1P and its analog fingolimod specifically activate S1PRs to initiate the Src/STAT3 signaling cascade. Phosphorylated STAT3 subsequently forms homodimers and undergoes nuclear translocation, where it significantly enhances transcriptional activity through direct binding to gamma-activated sequence elements in the promoter regions of *Slc7a11, Gpx4*, and *MnSOD* genes. This regulatory cascade ultimately upregulates the expression of SLC7A11, GPX4, and MnSOD proteins, which synergistically suppress ferroptosis and exert antioxidant effects by coordinately maintaining GSH homeostasis and scavenging lipid peroxides. (**Figure 10b**).

This study still has certain limitations. First, the research scope was confined to the acute phase of MI/R injury, primarily focusing on the regulatory effects of S1P and fingolimod on ferroptosis and oxidative stress in cardiomyocytes. We did not systematically evaluate their impacts on other cardiac cell types (including endothelial cells, fibroblasts, and immune cells), and the investigation of intercellular crosstalk mechanisms remains lacking. Second, the pharmacological intervention parameters for in vivo experiments (including dose gradients and time windows) were mainly based on previous research paradigms. We have not yet explored an optimized dose-response relationship system tailored to the specific characteristics of our experimental model.

## Conclusions

According to our research findings, both S1P and Fingolimod can modulate intracellular levels of SLC7A11, GPX4, and MnSOD in cardiomyocytes via the S1PRs/Src/STAT3 signaling pathway. This modulation enables them to exert anti-ferroptotic and antioxidant effects, effectively preserving mitochondrial morphology and function, and ultimately reducing MI/R induced cardiac injury. Among the S1PRs mediating these effects in cardiomyocytes, the S1PR1 receptor plays the most predominant protective role. These findings not only establish a novel molecular theoretical foundation for targeting ferroptosis in the prevention and treatment of myocardial ischemia-reperfusion injury but also provide critical experimental evidence to support the translational application of S1P and its analogs—particularly fingolimod—in clinical management of acute myocardial infarction, through revealing the dynamic regulatory mechanism governing STAT3 phosphorylation at Tyr705.

## Supplementary Material

Supplementary methods and figures.

## Abbreviations

ANOVA: Analysis of variance
ATP: Adenosine triphosphate
CCK-8: Cell counting kit-8
CK-MB: Creatine kinase-myocardial band
cTNT: Cardiac troponin T
DCFH-DA: 2',7'-dichlorodihydrofluorescein diacetate
DEGs: Differentially expressed genes
DHE: Dihydroethidium
DMEM: Dulbecco's modified Eagle medium
EF: Ejection fraction
FS: Fractional shortening
FBS: Fetal bovine serum
GPCR: G protein-coupled receptors
GPX4: Glutathione peroxidase 4
GSH: Glutathione
H/R: Hypoxia reoxygenation
JC-1: 5,5',6,6'-tetrachloro-1,1',3,3'-tetraethylbenzimidazolylcarbocyanine iodide staining
LAD: Left anterior descending
MMP: Mitochondrial membrane potential
MDA: Malondialdehyde
MI/R: Myocardial ischemia/reperfusion
MnSOD: Manganese superoxide dismutase
NRVMs: neonatal rat ventricular myocytes
p-STAT3: Phosphorylation signal transducer and activator of transcription 3
qRT-PCR: Quantitative reverse transcription polymerase chain reaction
ROS: Reactive oxygen species
Src: Tyrosine kinase family Src
SLC7A11: The cystine transporter solute carrier family 7 member 11
S1P: Sphingosine-1-phosphate
STAT3: Signal transducer and activator of transcription 3
S1PRs: Sphingosine-1-phosphate receptors
TEM: Transmission electron microscopy
TTC: Triphenyltetrazolium chloride
