# Supplementary material for: Activation of sphingosine-1-phosphate receptors can relieve myocardial ischemia-reperfusion injury by mitigating oxidative stress and ferroptosis in cardiomyocytes
Source: Int J Biol Sci. 2025 Jul 28;21(11):5079–96. doi: 10.7150/ijbs.107402 (PMC12374838; doi:10.7150/ijbs.107402)
Supplement: Supplementary file 1 — Supplementary methods and figures. [file ijbsv21p5079s1.pdf]

Original Article

Activation of sphingosine-1-phosphate receptors can relieve myocardial ischemia-reperfusion injury by mitigating oxidative stress and ferroptosis in cardiomyocytes

Xuan Xu[1,2]; Runqian Li[2]; Shengnan Li[2]; Qin Wei[1,2]; Fuchao Yu[1,2#]; Genshan Ma[1,2#]; Jiayi Tong [1,2#]

[1] Department of Cardiology, Zhongda Hospital, Southeast University, 87 Dingjiaqiao, Nanjing, P.R China 210009

[2] School of medicine, Southeast University, Nanjing 210009, P. R. China.

# Corresponding authors. E-mail: 101012514@seu.edu.cn (Fuchao Yu); magenshan@seu.edu.cn (Genshan Ma); tongjiayi@seu.edu.cn (Jiayi Tong)

Conflict of Interest Statement: The authors have no conflicts of interest to declare.

Supplementary Methods

1. Cell culture

H9C2 cells and AC16 were obtained from The Cell Bank of the Type Culture Collection of the Chinese Academy of Sciences. The cells were cultured in Dulbecco's modified Eagle medium (KGL1201-500, DMEM; Kaiji Biotech, Nanjing, China) containing 10% FBS (A5256701, Gibco BRL, Rockville, MD, USA), 100 U/ml penicillin, and 100 μg/ml streptomycin. Cell cultures were maintained in a 37°C incubator with 5% CO2, and subculturing was performed when the cell density reached 80%. Passaged cells were subsequently utilized for experiments.

2. Isolation of neonatal rat ventricular myocytes (NRVMs)

1-day-old SD rats were disinfected, followed by the isolation of their hearts, which were then washed three times with sterile phosphate-buffered saline (KGL2206-500, PBS; Kaiji Biotech) on ice. The left ventricles were minced using ophthalmic scissors. Subsequently, collagenase type II (1 mg/ml; 1148090, Sigma-Aldrich, USA) digestion was carried out at 37°C for 10 minutes, and the supernatant was collected. The supernatant was centrifuged at 200 rpm for 5 minutes to obtain the pellet. The process of digesting the cardiac tissue was repeated 8-10 times until a noticeable reduction in sedimentation was observed in the supernatant. Finally, the isolated cells were cultured in 10% DMEM medium, and cardiac myocytes were purified using the differential adhesion method. The purified NRVMs were cultured in 10% DMEM until reaching optimal cell confluence for subsequent experiments.

3. Cell experiment grouping strategy

The experiment investigating the optimal concentration of S1P was divided into five

groups. Control group: normal H9C2 cells; H/R group: H9C2 cells subjected to H/R treatment; H/R+S1P groups: treated with H/R followed by addition of S1P (40nM, 400nM, or 4μM; HY-108496, MedChemExpress, Shanghai, China) during reoxygenation for 12 hours. The dosage of S1P was determined based on previous studies (1, 2).

The experiment comparing the therapeutic effects of Ferrostatin-1 was structured into six groups. Group 1: normal H9C2 or NRVMs cells; Group 2: H9C2 or NRVMs cells subjected solely to H/R treatment; Group 3: H9C2 or NRVMs cells treated with S1P (400 nM) alone for 12 hours; Group 4: underwent H/R treatment followed by 12-hour treatment with S1P (400 nM) during reoxygenation for H9C2 or NRVMs cells; Group 5: treated exclusively with Ferrostatin-1 (50 μM, RM02804, ABclonal Technology, Wuhan, China) for H9C2 or NRVMs cells; Group 6: underwent H/R treatment and treat with Ferrostatin-1 (50 μM) during reoxygenation for H9C2 or NRVMs cells. The dosage of Ferrostatin-1 was determined based on previously (3).

In the experiment investigating the correlation between S1P and phosphorylated signal transducer and activator of transcription 3 (p-STAT3) expression, four groups were formed. Group 1: normal H9C2 or NRVMs cells; Group 2: underwent 12-hour treatment with S1P (400 nM) alone for H9C2 or NRVMs cells; Group 3: H9C2 or NRVMs cells subjected solely to H/R treatment; Group 4: underwent H/R treatment followed by 12-hour treatment with S1P (400 nM) during reoxygenation for H9C2 or NRVMs cells;

The experiment comparing MnSOD expression in cells treated with S1P and Ferrostatin-1 was divided into four groups. Group 1: normal H9C2 or NRVMs cells; Group 2: H9C2 or NRVMs cells subjected solely to H/R treatment; Group 3: underwent H/R treatment

followed by 12-hour treatment with S1P (400 nM) during reoxygenation for H9C2 or NRVMs cells; Group 4: underwent H/R treatment and were treated with Ferrostatin-1 (50 μM) during both hypoxia and reoxygenation for H9C2 or NRVMs cells.

In the experiment investigating the correlation between p-STAT3 and ferroptosis and ROS, five groups were established. Group 1: normal H9C2 cells; Group 2: H9C2 cells treated solely with Stattic (1 μM, HY-13818, MedChemExpress) for 3 hours; Group 3: H9C2 cells subjected solely to H/R treatment; Group 4: underwent H/R treatment followed by 12-hour treatment with S1P (400 nM) during reoxygenation; Group 5: H9C2 cells pretreated with Stattic (1 μM) for 3 hours before the end of hypoxia and then co-treated with Stattic and S1P for 12 hours during reoxygenation.

The experiment exploring S1P receptor inhibition was divided into four groups. H/R+S1P group: H9C2 cells subjected to H/R treatment followed by 12-hour treatment with S1P (400 nM) during reoxygenation; H/R+S1P+IR1 group: H9C2 cells pretreated with the S1PR1 inhibitor W146 (10 μM, HY-101395 ,MedChemExpress) for 3 hours before the end of hypoxia and cocultured with W146 and S1P for 12 hours during reoxygenation; H/R+S1P+IR2 group: H9C2 cells pretreated with the S1PR2 inhibitor JTE013 (10 μM, HY-100675, MedChemExpress) for 3 hours before the end of hypoxia and cocultured with JTE013 and S1P for 12 hours during reoxygenation; H/R+S1P+IR3 group: H9C2 cells pretreated with the S1PR3 inhibitor CAY10444 (10 μM, HY-119401, MedChemExpress) for 3 hours before the end of hypoxia and cocultured with CAY10444 and S1P for 12 hours during reoxygenation.

The experiments pertaining to Fingolimod were divided into two groups. H/R group:

H9C2 cells subjected to H/R treatment; H/R+ Fingolimod group – cells subjected to H/R modeling and treated with Fingolimod (100 nM, HY-11063, MedChemExpress) for 12 hours during reoxygenation. The dosage of Fingolimod was determined based on previous studies (4) (5).

4. Animal experiment grouping strategy

Mice were divided into four groups: 1. Sham group: mice underwent thoracotomy without ligation of the left anterior descending coronary artery. 2. MI/R group: mice received intraperitoneal injections of PBS during surgery and for three days postoperatively. 3. MI/R + S1P group: A murine MI/R model was established, in which mice received intraperitoneal injections of S1P (6mg/kg) during surgery and for three days postoperatively, with a dosing regimen of three times per day. The dosage of S1P administered was based on previous studies (6). 4. MI/R + Fingolimod group: A murine MI/R model was established, in which mice received tail vein injections of Fingolimod (1 mg/kg/day) during surgery and for three days postoperatively. The dosage of Fingolimod administered was based on previous studies (7).

5. Cell viability

Cell viability was assessed using the Cell Counting Kit-8 (CCK-8; KGA9305-1000, Kaiji Biotech) assay. In brief, H9C2 cells (5×10^4 cells per well) were seeded in a 96-well plate and cultured until reaching 80% confluence. Following the aforementioned treatments, CCK-8 solution (10 μl; Kaiji Biotech) was added to the H9C2 cells, and the cells were incubated at 37°C for 1 hour. The absorbance intensity of each well was measured at 450/650 nm using a multimode microplate reader (Infinite M200 PRO, Tecan Instruments,

USA). Cell viability was calculated using the following formula: cell viability (%) = (measurement value - blank value)/(control value - blank value) × 100%.

6. Cell ROS and mitochondrial ROS assay

Intracellular and mitochondrial ROS levels were measured using 2',7'-dichlorodihydrofluorescein diacetate (DCFH-DA; S0033, Beyotime, Nantong, China) and MitoSOX™ (M36008; Thermo Fisher Scientific) Red fluorescent probes, respectively, in accordance with the manufacturer's protocols. Briefly, cells from each experimental group were incubated with DCFH-DA and MitoSOX™ probes under standardized conditions. The cardiac tissue levels of ROS were assessed using dihydroethidium (DHE; S0064, Beyotime) staining. In brief, mice were euthanized, and the hearts were promptly excised and frozen for sectioning. Subsequently, staining procedures were conducted according to the manufacturer's instructions, followed by imaging using an inverted fluorescence microscope (TS100 / TS100-F, Nikon Co., Ltd., Japan).

7. Mitochondrial membrane potential (MMP) assay

Cells from different experimental groups and ventricular tissues frozen sections were stained with 5,5',6,6'-tetrachloro-1,1',3,3'-tetraethylbenzimidazolylcarbocyanine iodide staining (JC-1) dye (C2005, Beyotime) (37°C for 30 minutes) following the manufacturer's instructions. After the incubation period, the cells were washed with PBS. Subsequently, fluorescence images were captured using an inverted fluorescence microscope (TS100/TS100-F). ImageJ software was utilized for data analysis.

8. Transmission electron microscopy (TEM)

H9C2 cells and ventricular tissues were collected from each group and fixed in 3%

glutaraldehyde at 4°C overnight. The samples were postfixed in 1% osmium tetroxide for 2 hours and dehydrated with a gradient ethanol series. Subsequently, 75-nm sections were prepared and stained with 2% uranyl acetate and citric acid, and finally, mitochondrial morphology was observed using transmission electron microscopy (TEM, Hitachi, HT7800/HT7700, JPN). Flameng score: 0 points - Normal structure, 1 point - The structure is basically normal, but the matrix particles are lost (slight swelling, matrix density is reduced, cristae separation), 2 points - Mitochondrial swelling (reduced matrix density, cristae separation); matrix is transparent, cristae is not broken, 3 points - Mitochondrial cristae rupture, matrix coagulation (severe swelling), 4 points - Mitochondrial cristae rupture, the integrity of the inner and outer membranes disappears, and becomes vacuolated (severe swelling, rupture of the inner and outer membranes).

9. Measurement of GSH，Fe^{2+} and MDA

The levels of GSH (ml076450, MLbio, Shanghai, China), Fe^{2+} (ab83366, Abcam, Shanghai, China), and MDA (ml094962, MLbio) were quantified in cells and ventricular tissues from each experimental group using commercial assay kits according to the manufacturers' instructions. The optical density (OD) values for each well were measured at the appropriate wavelength using an Infinite M200 PRO multimode microplate reader. Subsequently, the obtained measurements were used to calculate the GSH, Fe^{2+}, and MDA concentrations.

10. Western blot:

Total protein from cells and ventricular tissues was extracted using RIPA lysis buffer (P0013B, Beyotime), and the protein concentration was determined with a BCA protein

assay kit (P0009, Beyotime). Protein samples were separated on a 12% SDS–PAGE gels and subsequently transferred onto PVDF membranes (IPVH00010, Millipore, Billerica, MA, USA). PVDF membranes were blocked with 5% nonfat milk powder for 1 hour. The membranes were then incubated overnight at 4°C with primary antibodies, including anti-SLC7A11 (1:1000, 26864-1-AP, Proteintech, Wuhan, China), anti-GPX4 (1:1000, ab125066, Abcam), anti-S1PR1 (1:1000, 55133-1-AP, Proteintech), anti-S1PR2 (1:1000, ab306562, Abcam), anti-S1PR3 (1:1000, 55204-1-AP, Proteintech), anti-Lamin B (1:2000, 12987-1-AP, Proteintech), anti-NaKATPase (1:2000, 14418-1-AP, Proteintech), anti-MnSOD (1:1000, ab68155, Abcam), anti-p-STAT3 (1:1000, ab267373, Abcam), anti-STAT3 (1:1000, 10253-2-AP, Proteintech), Src (1:1000, ab109381, Abcam), p-Src (1:1000, ab185617, Abcam), GAPDH (1:1000, 10494-1-AP, Proteintech)and anti-β-actin (1:2000, 20536-1-AP, Proteintech). Following incubation, the membranes were washed three times with TBST (ST671, Beyotime) and incubated with horseradish peroxidase-conjugated secondary antibodies (A0208, Beyotime) at room temperature for 1 hour. Finally, protein bands were visualized using an ECL chemiluminescence kit (P0018S, Beyotime) and a gel imaging system (Tanon, Shanghai, China).

11. RT-PCR

Total RNA was isolated using TRIzol (R0016, Beyotime), followed by cDNA synthesis using the PrimeScript RT Master Mix Kit (RR036, Takara, Shiga, Japan). TB Green Premix Ex Taq (Takara) was used for the PCR reactions, and PCR was performed on the Mx3000p (Agilent Technologies, California, USA) system. The primer sequences used for RT-PCR analysis are as follows: Mouse *Gpx4*: forward 5′ -

GATGGAGCCCATTCCTGAACC -3′, reverse 5′ - CCCTGTACTTATCCAGGCAGA - 3′; Rat *Gpx4*: forward 5′ - GGACCTGCCGTGCTATCT -3′, reverse 5′ - GGCCTCTGGACCTTCCTC -3′; Mouse *Slc7a11*: forward 5′ - GGCACCGTCATCGGATCAG -3′, reverse 5′ - CTCCACAGGCAGACCAGAAAA -3′; Rat *Slc7a11*: forward 5′ - GCAAGCATAA GCATCAGGTA -3′, reverse 5′ - TAAGTTCTCCAGGCATCCA -3′; Mouse *MnSOD*: forward 5′ - CAGACCTGCCTTACGACTATGG -3′, reverse 5′ - CTCGGTGGCGTTGAGATTGTT - 3′; Rat *MnSOD*: forward 5′ - ATTAACGCGCAGATCATGCA -3′, reverse 5′ - CCTCGGTGACGTTCAGATTGT -3′.

12. TTC staining

All groups of mice were euthanized 24 hours postoperatively, and the hearts were rapidly excised, embedded in OCT, and placed in a -20°C freezer for 30 minutes. Subsequently, the ischemic area was sectioned at a thickness of 1mm and then incubated in triphenyltetrazolium chloride (TTC; T8877, Sigma) staining solution for 15 minutes at 37°C.

13. Echocardiogram

At 3 days postoperatively, two-dimensional guided M-mode echocardiography (Vevo 2100, Canada) was employed to assess mouse cardiac function. In brief, mice were continuously anesthetized with 2% isoflurane, and the MS400 probe was used to obtain the parasternal long-axis view to measure various parameters, including left ventricular ejection fraction (EF) and fractional shortening (FS).

14. Luciferase assays

HEK293T cells were seeded into a 48-well culture plate and cultured in DMEM medium containing 10% FBS. Once the cell density reached the experimental requirements, the original medium was discarded, and FBS-free DMEM was added. The plasmids pcDNA3.1-Stat3, pGL3-Basic, pGL3-MnSOD-WT, pGL3-MnSOD-MUT, and pRL-TK were transfected into HEK293T cells using Lipofectamine 3000 (Thermo Fisher Scientific, Massachusetts, USA) according to the experimental design, establishing experimental and control groups. The cells were incubated in a cell culture incubator for 48 hours, after which they were lysed. Finally, the Luciferase Assay Reagent II (Promega, Wisconsin, USA) was mixed with the cell lysate, and the enzyme activity of Firefly luciferase was measured using a microplate reader. After recording the data, Stop & Glo® Reagent (Promega) was quickly added to detect the enzyme activity of Renilla luciferase.

15. Extraction of membrane proteins and nuclear proteins

Membrane proteins and nuclear proteins from H9C2 cells and cardiomyocytes were extracted using the membrane protein extraction kit (P0033, Beyotime) and nuclear protein extraction kit (P0028, Beyotime) according to the manufacturer's instructions, and the extracts were subsequently used for Western blot experiments.

16. ChIP-qPCR

AC16 cells were treated with Stattic or DMSO and cultured in the medium until they reached 70-80% confluence. To cross-link proteins to DNA, cells were treated with 1% formaldehyde for 10 minutes at room temperature, followed by quenching the reaction with 0.125 M glycine for 5 minutes. Subsequently, cells were washed with PBS and lysed in a lysis buffer. The lysate was sonicated, then diluted in immunoprecipitation buffer, the

IgG and STAT3 (Proteintech) antibody were added, followed by overnight incubation at 4°C with rotation. Protein A/G beads (P2083, Beyotime) were added, and the mixture was incubated for 2 hours. Washing the beads with wash buffers, the immunoprecipitated DNA was eluted, and cross-linking was reversed by heating at 65°C for several hours. Then, the DNA was purified with a GeneJET Gel Extraction Kit (K0691, Thermo Fisher Scientific). For qPCR, the purified DNA was used as a template in reactions with specific primers targeting the region of interest. Genomic DNA enrichment was calculated as the percentage of input. The primer sequences for *MnSOD* are as follows: Forward primer GAGTTGGTACGGCCCGAAG, Reverse primer AACCAAAACTCAGGGGCAGG.

17. Bioinformatics analysis

Binding site prediction: Access the NCBI-Gene database (https://www.ncbi.nlm.nih.gov/gene/) to find promoter information for *Slc7a11*, *Gpx4*, and *MnSOD*. Search for transcription factor STAT3 on the JASPAR website (https://jaspar.genereg.net/), set a threshold of 85%, and predict binding sites using the promoters of *Slc7a11*, *Gpx4*, and *MnSOD*, prioritizing high-score prediction sites.

ChIP-seq analysis: Access the GEO datasets (https://www.ncbi.nlm.nih.gov/geo/; GSE117164, GSE212076) and utilize IGV software to examine and analyze the signal enrichment for the promoters of *Slc7a11*, *Gpx4*, and *MnSOD*, as well as for STAT3.

STAT3-ROS related differential gene analysis: Download the GEO dataset (GSE184649), set Log2FC > 0.43 and $P < 0.05$ to identify differential genes, and use the VLOOKUP function to find ROS-related genes in the DEGs, with their expression visualized using RStudio.

Correlation analysis of mRNA in human cardiac tissue: Visit the GEPIA database (http://gepia.cancer-pku.cn) to conduct Pearson correlation analysis of *Stat3* mRNA expression with *Slc7a11*, *Gpx4*, and *MnSOD* mRNA in human left ventricle tissue, and create visual representations.

18. Cell ATP measurement

H9C2 cells were cultured to experimental confluency, treated as described for the experimental groups, and subsequently lysed. Cellular ATP levels were then quantified using a commercial ATP assay kit (S0026, Beyotime) according to the manufacturer's protocols. Briefly, a standard curve was established with the ATP detection working solution, followed by systematic measurement of ATP levels in all treatment groups.

Supplementary Figures

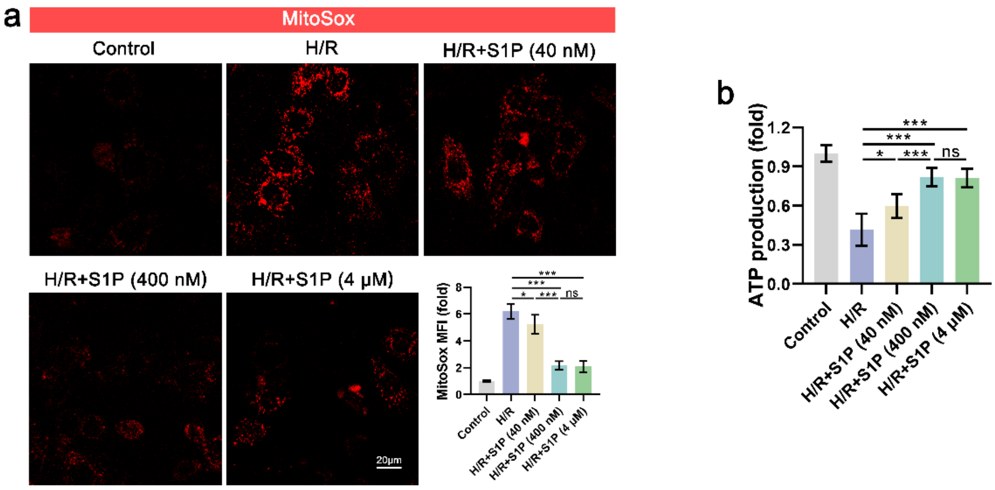

Figure S1 S1P attenuates H/R-induced mitochondrial oxidative stress, and ATP levels in H9C2 cells. a. Measurement of mitochondrial ROS levels in H9C2 cells using MitoSOX™ Red fluorescent probe and analysis of mean fluorescence intensity (MFI); n=6. b. Quantitative analysis of ATP production in various groups of H9C2 cells; n=6. All data are means ±

standard deviations. Statistical analysis involved one-way ANOVA followed by Tukey's post-hoc test. Lines indicate comparisons between samples, and asterisks denote statistical significance (**P* < 0.05, ***P* < 0.01, ****P* < 0.001).

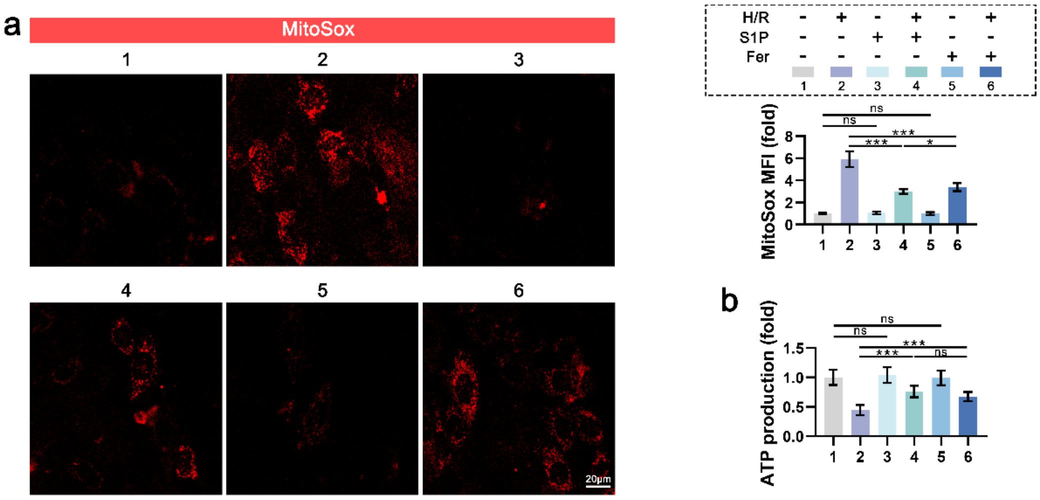

Figure S2 S1P and Ferrostatin-1 can attenuate H/R-induced mitochondrial oxidative stress, and ATP production in H9C2 cells. a. Mitochondrial ROS levels in H9C2 cardiomyocytes treated with S1P or Ferrostatin-1 were measured using MitoSOX™ Red fluorescent probe, with subsequent analysis of mean fluorescence intensity; n=6. b. ATP levels were quantified in S1P or Ferrostatin-1 treated H9C2 cardiomyocytes. All data are means ± standard deviations. Statistical analysis involved one-way ANOVA followed by Tukey's post-hoc test. Lines indicate comparisons between samples, and asterisks denote statistical significance (**P* < 0.05, ***P* < 0.01, ****P* < 0.001).

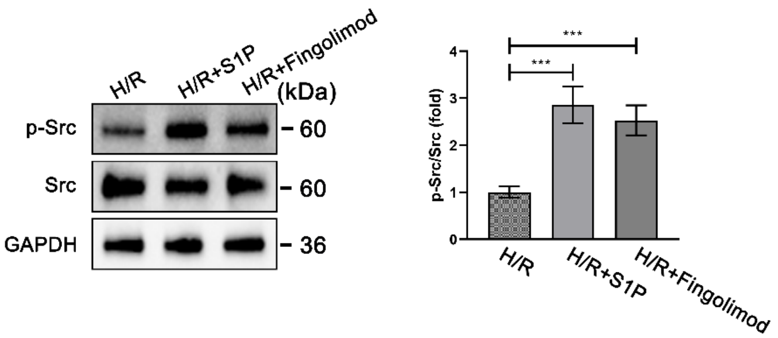

Figure S3 S1P and fingolimod activate Src signaling. Representative Western blots and quantification of p-Src and Src levels in H9C2 cells. GAPDH served as the loading control. n = 6. All data are means ± standard deviations. Statistical analysis involved one-way ANOVA followed by Tukey's post-hoc test. Lines indicate comparisons between samples, and asterisks denote statistical significance (*P < 0.05, **P < 0.01, ***P < 0.001).

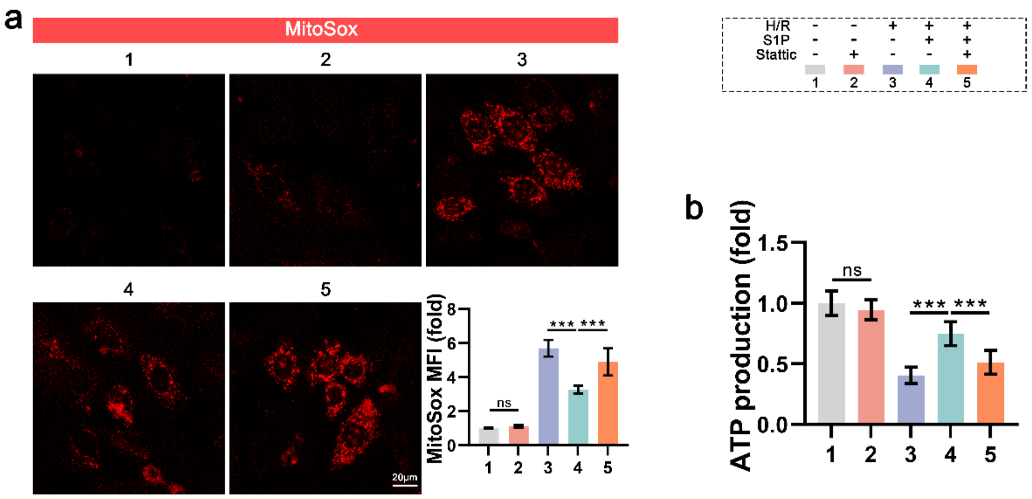

Figure S4 Stattic inhibits both S1P's antioxidant effects against mitochondrial oxidative damage and its protective actions on mitochondrial function. a. Mitochondrial ROS levels in H9C2 cardiomyocytes treated with S1P or Stattic were measured using MitoSOX™ Red fluorescent probe, with subsequent analysis of mean fluorescence intensity; n=6. b. ATP levels were quantified in S1P or Stattic treated H9C2 cardiomyocytes. All data are means ± standard deviations. Statistical analysis involved one-way ANOVA followed by Tukey's post-hoc test. Lines indicate comparisons between samples, and asterisks denote statistical significance (*P < 0.05, **P < 0.01, ***P < 0.001).

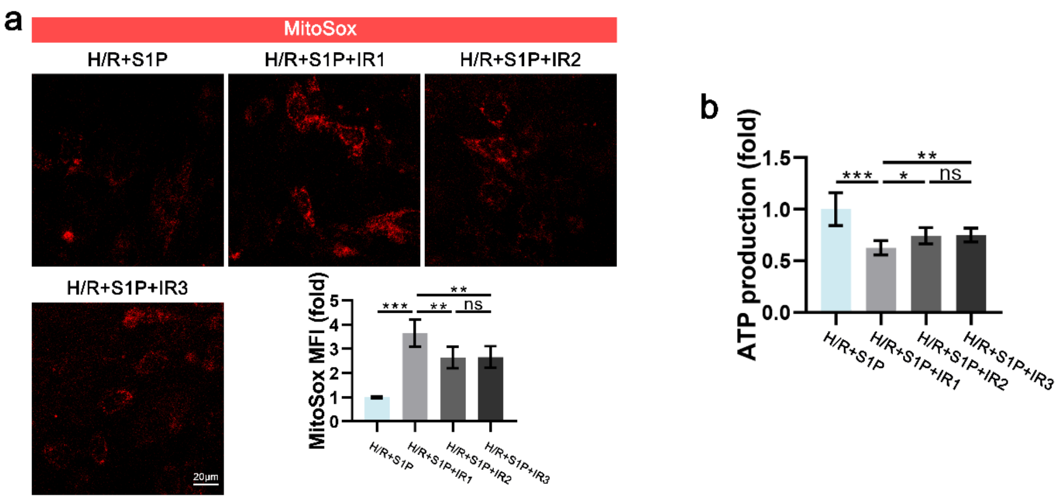

Figure S5 Inhibition of S1PR1/2/3 abolishes the therapeutic effects of S1P on mitochondrial oxidative stress and functional homeostasis in cardiomyocytes. a. Mitochondrial ROS levels in H9C2 cardiomyocytes treated with IR1/2/3 were measured using MitoSOX™ Red fluorescent probe, with subsequent analysis of mean fluorescence intensity; n=6. b. ATP levels were quantified in IR1/2/3 treated H9C2 cardiomyocytes. All data are means ± standard deviations. Statistical analysis involved one-way ANOVA followed by Tukey's post-hoc test. Lines indicate comparisons between samples, and asterisks denote statistical significance (**P* < 0.05, ***P* < 0.01, ****P* < 0.001). IR1= W146; IR2= JTE013; IR3=CAY10444.

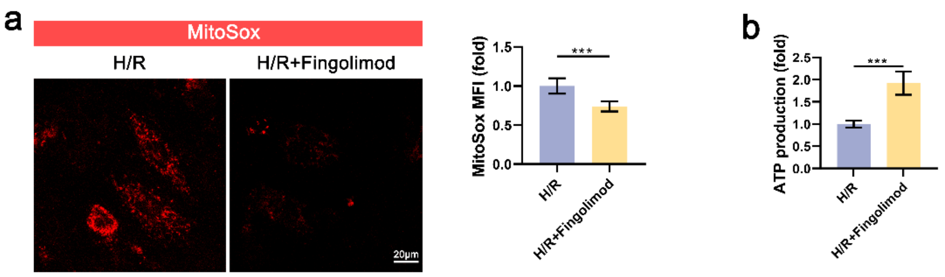

Figure S6 Fingolimod can attenuate H/R-induced mitochondrial oxidative stress, and ATP production in H9C2 cells. a. Mitochondrial ROS levels in H9C2 cardiomyocytes treated with Fingolimod were measured using MitoSOX™ Red fluorescent probe, with subsequent

analysis of mean fluorescence intensity; n=6. b. ATP levels were quantified in Fingolimod treated H9C2 cardiomyocytes. All data are means ± standard deviations. Statistical analysis involved one-way ANOVA followed by Tukey's post-hoc test. Lines indicate comparisons between samples, and asterisks denote statistical significance (*$P < 0.05$, **$P < 0.01$, ***$P < 0.001$).

References

1. Vessey DA, Li L, Honbo N, Karliner JS. Sphingosine 1-phosphate is an important endogenous cardioprotectant released by ischemic pre- and postconditioning. American journal of physiology Heart and circulatory physiology. 2009;297(4):H1429-35.
2. Wang Y, Wang D, Zhang L, Ye F, Li M, Wen K. Role of JAK-STAT pathway in reducing cardiomyocytes hypoxia/reoxygenation injury induced by S1P postconditioning. European Journal of Pharmacology. 2016;784:129-36.
3. Miyamoto HD, Ikeda M, Ide T, Tadokoro T, Furusawa S, Abe K, et al. Iron Overload via Heme Degradation in the Endoplasmic Reticulum Triggers Ferroptosis in Myocardial Ischemia-Reperfusion Injury. JACC Basic to translational science. 2022;7(8):800-19.
4. Muñoz JP, Sànchez-Fernàndez-de-Landa P, Diarte-Añazco EMG, Zorzano A, Blanco-Vaca F, Julve J. FTY720-P, a Biased S1PR Ligand, Increases Mitochondrial Function through STAT3 Activation in Cardiac Cells. International journal of molecular sciences. 2023;24(8).
5. Hofmann U, Burkard N, Vogt C, Thoma A, Frantz S, Ertl G, et al. Protective effects of sphingosine-1-phosphate receptor agonist treatment after myocardial ischaemia-reperfusion. Cardiovascular research. 2009;83(2):285-93.
6. Chen K, Wang Z, Liu C, Yang X, Jiang J. Sphingosine-1-phosphate Attenuates Endoplasmic Reticulum Stress-induced Cardiomyocyte Apoptosis Through Sphingosine-1-phosphate Receptor 1. Archives of medical research. 2022;53(6):562-73.
7. Santos-Gallego CG, Vahl TP, Goliasch G, Picatoste B, Arias T, Ishikawa K, et al. Sphingosine-1-Phosphate Receptor Agonist Fingolimod Increases Myocardial Salvage and Decreases Adverse Postinfarction Left Ventricular Remodeling in a Porcine Model of Ischemia/Reperfusion. Circulation. 2016;133(10):954-66.
